# Theoretical insights into Cu-mediated electronic modulation in FeCoNi-based multimetallic catalysts for polysulfide conversion

**DOI:** 10.1039/d6ra06165f

**Published:** 2026-09-18

**Authors:** Xin Guo, Jianshuang Wei, Xiuju Song, Jingyu Sun, Yixiong Feng

**Affiliations:** a State Key Laboratory of Public Big Data, Guizhou University Guiyang 550025 Guizhou Province China fyxtv@zju.edu.cn; b College of Mechanical Engineering, Guizhou University Guiyang 550025 Guizhou Province China; c State Key Laboratory of Fluid Power & Mechatronic Systems, Zhejiang University Hangzhou 310058 China; d College of Energy, Soochow Institute for Energy and Materials Innovations, Key Laboratory of Advanced Carbon Materials and Wearable Energy Technologies of Jiangsu Province, Soochow University Suzhou China

## Abstract

FeCoNi is a widely used multimetallic framework for lithium–sulfur electrocatalysis, yet the mechanistic contribution of Cu remains incompletely resolved. Here, calculations on representative FeCoNi and dilute single-CuFe_8_Co_10_Ni_13_Cu_1_ models are employed to elucidate how local Cu incorporation regulates polysulfide adsorption, sulfur-reduction thermodynamics and interfacial charge redistribution. The calculations reveal that Cu incorporation selectively strengthens soluble long-chain Li_2_S_6_ adsorption, decreases the limiting 
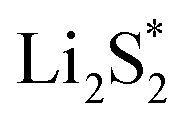
 to Li_2_S* free-energy change from 1.07 to 1.01 eV and induces pronounced local charge redistribution within the cooperative Fe/Co/Ni/Cu environment. A batch-matched FeCoNi@CP control further validates the catalytic modulation effect of Cu. Modulus-weighted Li_2_S_6_ symmetric-cell fitting yields *R*_ct_ values of 10.74, 38.23 and 144.89 Ω for FeCoNiCu@CP, FeCoNi@CP and pristine CP, respectively, while matched variable-scan-rate CV measurements demonstrate enhanced low-potential cathodic kinetics for FeCoNiCu@CP. At 2C, FeCoNiCu@CP delivers higher and more stable early-stage electrochemical performance than FeCoNi@CP, achieving 739.0 *versus* 608.0 mA h g^−1^ at cycle 10 and 663.9 *versus* 621.8 mA h g^−1^ at cycle 100. Element-resolved Bader charge analysis further suggests that Fe and Ni act as the main charge-transfer reservoirs, Co participates in cooperative charge donation, and Cu serves as a highly responsive local electronic regulator during Li_2_S_4_/Li_2_S_6_ adsorption. These findings indicate that Cu does not function as an isolated dominant active center, but electronically modulates the FeCoNi-based multimetallic surface to balance polysulfide anchoring and conversion. These results demonstrate that Cu incorporation promotes interfacial electron regulation and accelerates polysulfide conversion through a local electronic modulation effect rather than acting as an isolated dominant active site.

## Abstract 2

Cu acts as a local electronic modulator in FeCoNi-derived catalysts, enhancing early polysulfide-conversion kinetics without improving long-term retention.

## Graphical abstract

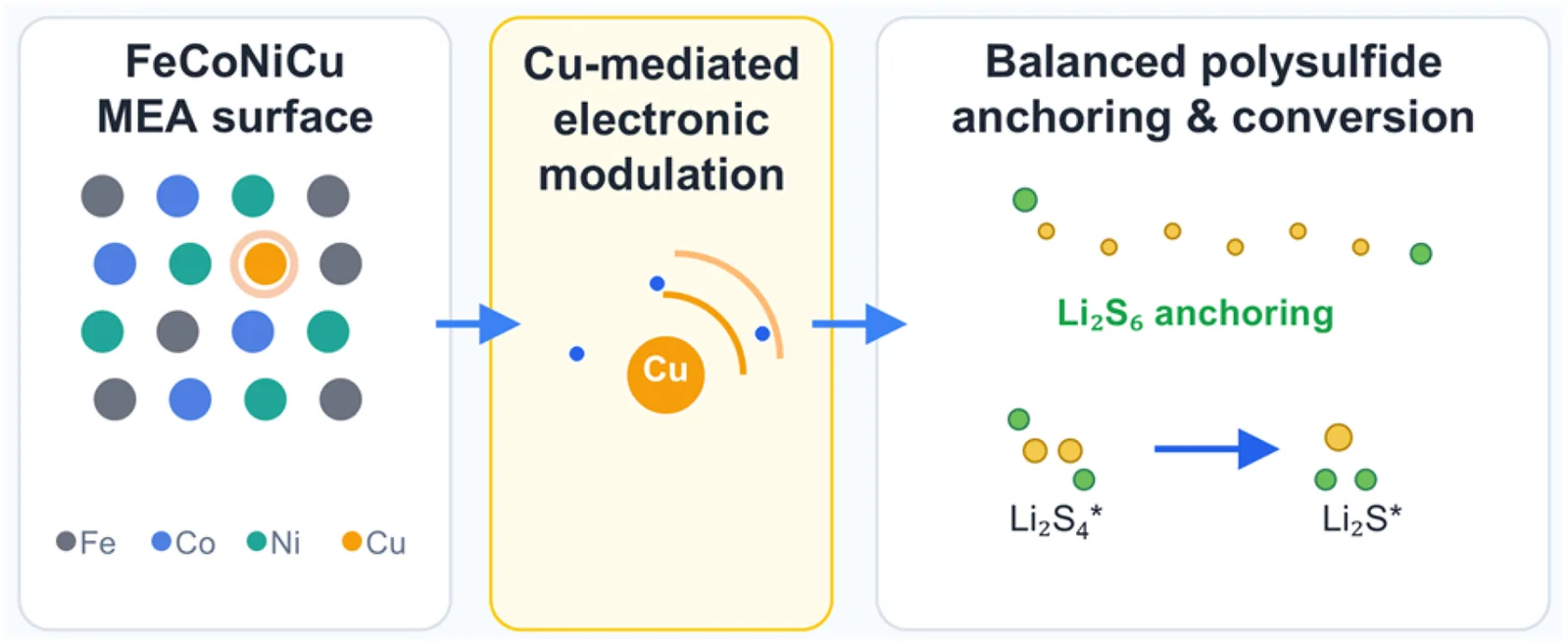

## Introduction

Lithium–sulfur (Li–S) batteries are deemed strong contenders for next-generation energy-storage systems because sulfur offers a high theoretical capacity, high theoretical energy density, natural abundance and low cost.^1–3^ Nevertheless, their practical implementation is severely impeded by the multistep conversion chemistry of solid S_8_, soluble long-chain lithium polysulfides and insoluble Li_2_S_2_/Li_2_S products. The dissolution and migration of lithium polysulfides (LiPSs) trigger polysulfide shuttle effect, active-material loss and parasitic reactions on the Li-metal anode, while sluggish Li_2_S nucleation/decomposition and the insulating nature of sulfur species lead to large polarization and rapid capacity decay under high current densities.^4–6^

Considerable efforts have therefore been devoted to constructing sulfur hosts and catalytic interlayers that can regulate LiPSs adsorption and conversion. Defect-rich carbon frameworks, polar compounds, polymeric immobilization layers, heterostructures and electrocatalytic materials have been developed to strengthen chemical anchoring, accelerate stepwise sulfur redox reactions and direct Li_2_S deposition.^7–14^ These advances establish a central design principle for Li–S catalysts: an effective host should capture soluble LiPSs strongly enough to suppress outward diffusion, yet avoid excessive binding that would hinder subsequent conversion and product release.

High- and medium-entropy alloys offer an attractive route to this adsorption-conversion balance because their disordered solid-solution lattices contain diverse local atomic configurations, tunable electronic structures and multiple ensemble sites.^15–20^ Recent Li–S studies demonstrate that compositionally complex catalysts can promote polysulfide conversion through broad adsorption-energy distributions, d-band/orbital modulation, relay catalysis and interfacial electron-density regulation. Across this literature, FeCoNi repeatedly appears as a chemically active base framework, whereas Cu is often introduced as an additional element in FeCoNi-containing alloys. However, whole-composition performance comparisons cannot determine whether Cu is a dominant active center, a passive entropy contributor, or an electronic regulator of neighboring Fe/Co/Ni sites.^21–31^

Fe, Co and Ni are representative 3d transition metals whose partially filled d states can interact with sulfur-containing intermediates. Fe-containing sites can provide polar interaction with sulfur species, Co supplies additional redox-responsive coordination environments, and Ni contributes to local electron-density and kinetic regulation. FeCoNi is therefore used here as the reference framework. Cu was selected a priori—not because it can be identified as a universally optimal fourth element from the TDOS integral alone, but because Fe–Co–Ni–Cu is an established multimetallic family and FeCoNiCu_0.5_ has been reported as a representative Cu-containing FeCoNi multimetallic composition.^32^ However, the mechanistic rationale for introducing Cu into this FeCoNi-based framework has not been fully clarified. The scientific question is consequently not whether Cu is universally superior to every possible fourth element, but why Cu remains mechanistically useful despite its comparatively modest intrinsic polysulfide affinity.^31^

In this work, we establish a computation-centered framework to determine why FeCoNiCu is effective for polysulfide regulation and why Cu is a rational addition to the FeCoNi framework. FeCoNi and FeCoNiCu are evaluated using the same lattice, slab, adsorbate and electronic-structure protocol. Adsorption configurations, adsorption-energy differences, sulfur-reduction free-energy profiles, near-Fermi electronic states and element-resolved Bader charge redistribution form a hierarchy of mutually supporting evidence. The strength of the mechanistic conclusion therefore lies in the convergence of several independent computational descriptors, rather than in any single adsorption energy or DOS value.

To connect the representative-model mechanism with material reality, FeCoNiCu@CP and a batch-matched Cu-free FeCoNi@CP control were prepared with the same nominal total-metal input and downstream processing.^32^ Li_2_S_6_ symmetric-cell EIS, matched full-cell variable-scan-rate CV and 2C cycling provide direct within-study experimental comparisons. The EIS, CV and early-cycle capacity results support a Cu-associated kinetic modulation trend, whereas the 1000-cycle comparison prevents this trend from being overstated as superior long-term retention. The adsorption, free-energy, DOS and Bader calculations supply a local atomistic interpretation within the explicitly stated model boundaries.

## Results and discussion

### Controlled construction of FeCoNi and FeCoNiCu MEA models

The retained FeCoNi candidate set comprised 20 randomly/probabilistically selected near-equiatomic configurations with the same Fe_10_Co_11_Ni_11_ composition, whereas all 20 Cu-containing configurations had the fixed Fe_8_Co_10_Ni_13_Cu_1_ composition. Because high-/medium-entropy alloys are intrinsically disordered, these finite cells were used to sample possible local atomic arrangements rather than to reproduce a full statistical ensemble. Raw total energies were compared only within each fixed-composition set. Configuration 5 is the lowest-energy structure within the fixed Fe_10_Co_11_Ni_11_ set and was used to construct the representative Fe_20_Co_22_Ni_22_ slab; configuration 5 is also the lowest within the fixed Fe_8_Co_10_Ni_13_Cu_1_ group and was expanded to Fe_16_Co_20_Ni_26_Cu_2_. These models therefore probe a near-equiatomic FeCoNi surface and a dilute local Cu perturbation, respectively, rather than an equimolar precursor replica or a statistical ensemble average (Fig. S1 and S2).

The optimized bulk configurations were further expanded by a factor of two to construct surface models with a 15 Å vacuum layer to avoid interactions between periodic images. Considering that exposed (111) fringes have been observed in FeCoNiCu_0.5_ and that the (111) surface is widely used as a representative close-packed facet for FCC high-entropy alloys,^32,34^ the (111) surface was selected as a common representative orientation for both FeCoNi and FeCoNiCu models. This consistent surface platform enables comparison of sulfur-species adsorption and electronic interactions; it is not presented as proof that (111) is the unique equilibrium facet of the exact compositions studied here.

**Fig. 1:**
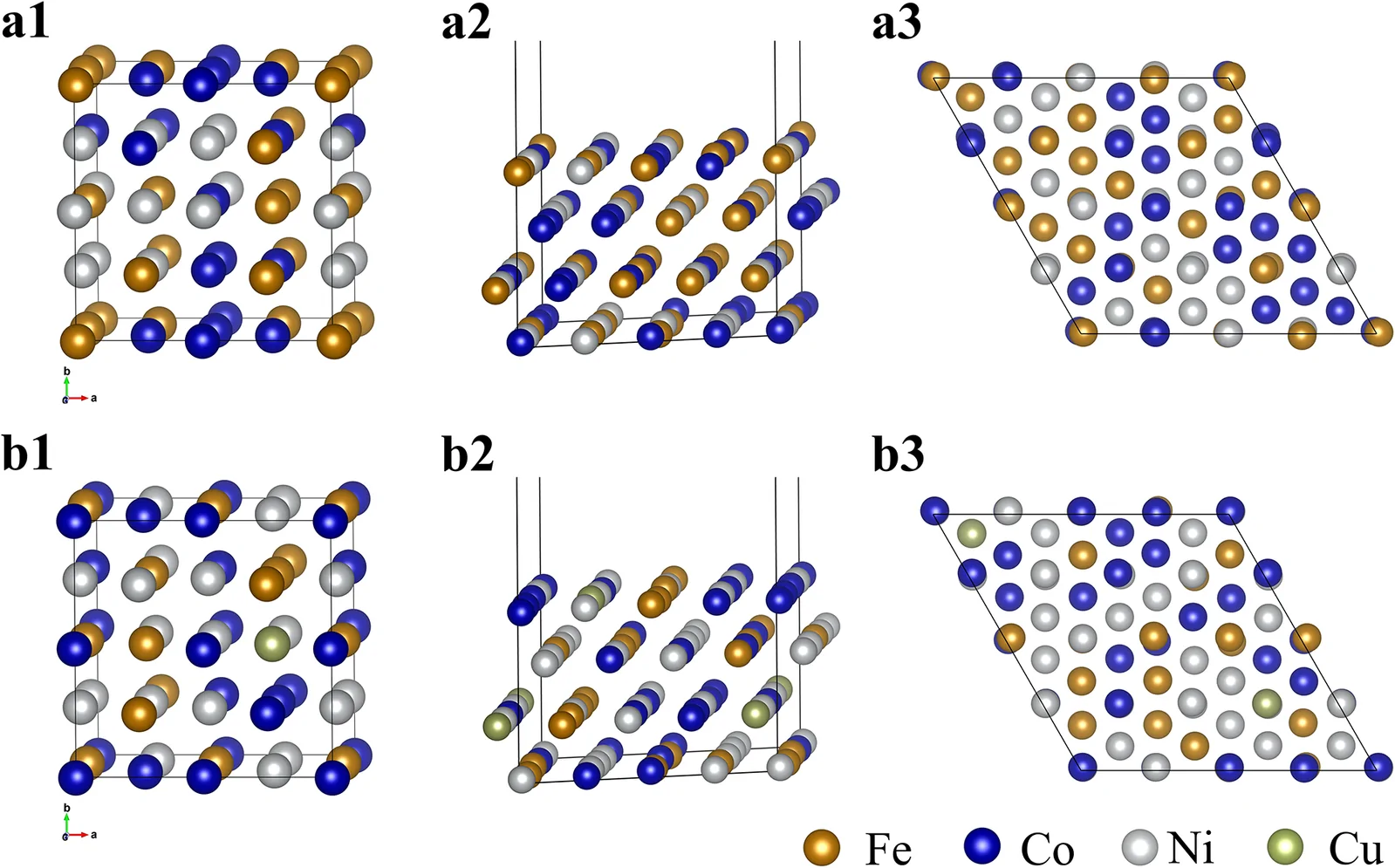
Bulk structures and side and top views of the constructed (111) slab models for the ternary FeCoNi MEA (a1–a3) and the Cu-incorporated FeCoNiCu MEA (b1–b3). Panels a1 and b1 show the bulk models, a2 and b2 show the side views, and a3 and b3 show the top views.

As shown in Fig. 1, both FeCoNi and FeCoNiCu expose chemically heterogeneous surface environments rather than a single idealized active site. In such multimetallic surfaces, Li and S atoms can interact with different neighboring metal combinations during adsorption. The FeCoNiCu surface further introduces Cu-containing local ensembles, which are expected to adjust the spatial distribution of electron donation and acceptance.^33^ Therefore, the model comparison is not intended to identify one isolated Cu site as the only active center; instead, it evaluates how Cu changes the collective electronic response of the FeCoNi-based MEA surface.

**Fig. 2:**
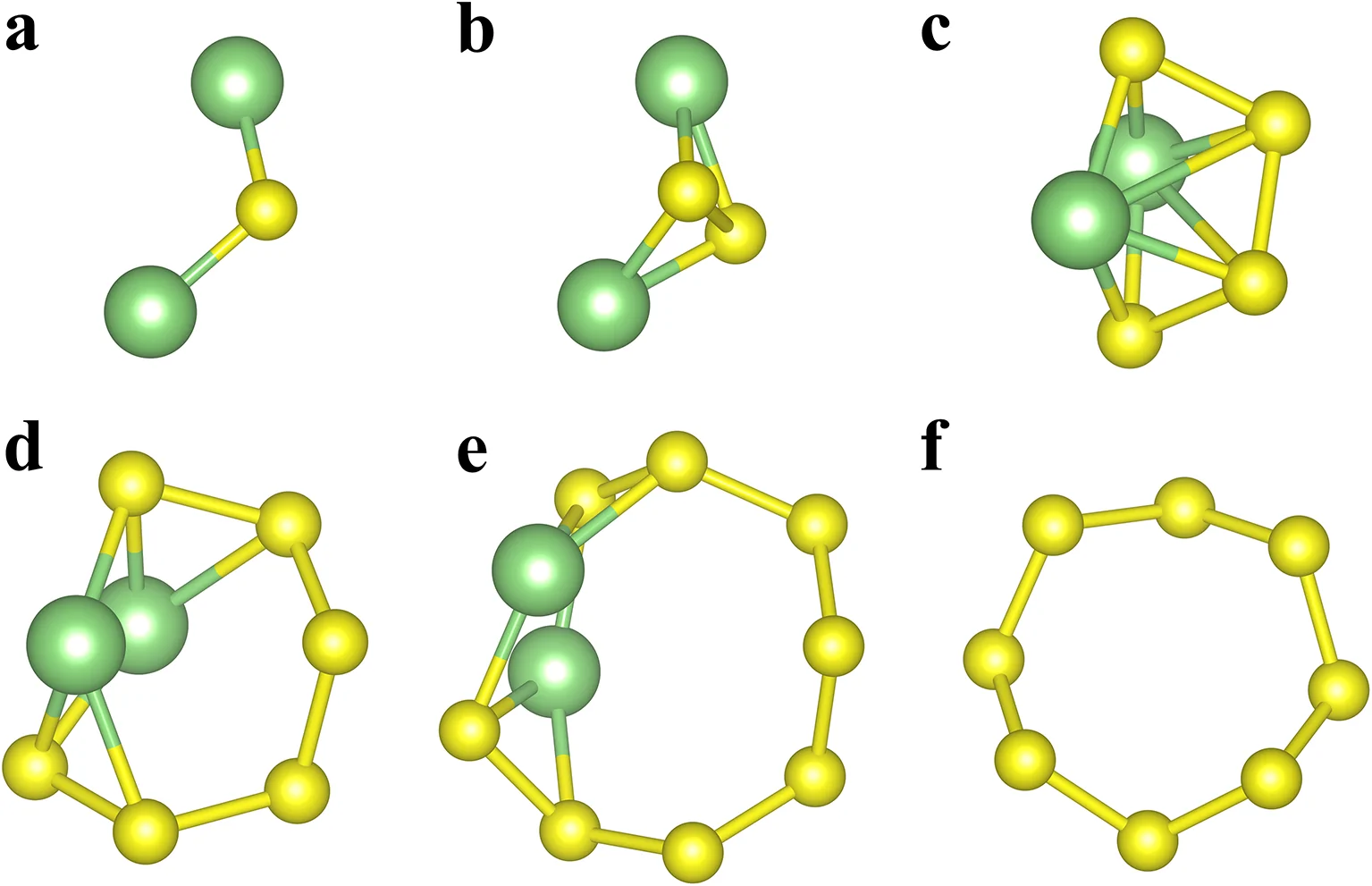
Optimized isolated structures of (a) Li_2_S, (b) Li_2_S_2_, (c) Li_2_S_4_, (d) Li_2_S_6_, (e) Li_2_S_8_, and (f) S_8_. Yellow and green spheres denote S and Li atoms, respectively.

Before adsorption calculations, isolated sulfur species were optimized to provide consistent reference states for adsorption-energy and reaction-free-energy analysis. Fig. 2 shows the optimized geometries of Li_2_S, Li_2_S_2_, Li_2_S_4_, Li_2_S_6_, Li_2_S_8_ and S_8_. These species cover representative molecular/ionic intermediates involved in Li–S discharge and charge, from ring-like elemental sulfur and soluble long-chain LiPSs to short-chain and insoluble discharge products. Using the same reference set for both alloy surfaces ensures that adsorption-energy differences reflect catalyst-surface effects rather than inconsistencies in isolated adsorbate structures.

### Cu incorporation regulates LiPS adsorption on FeCoNi-based MEA surfaces

**Fig. 3:**
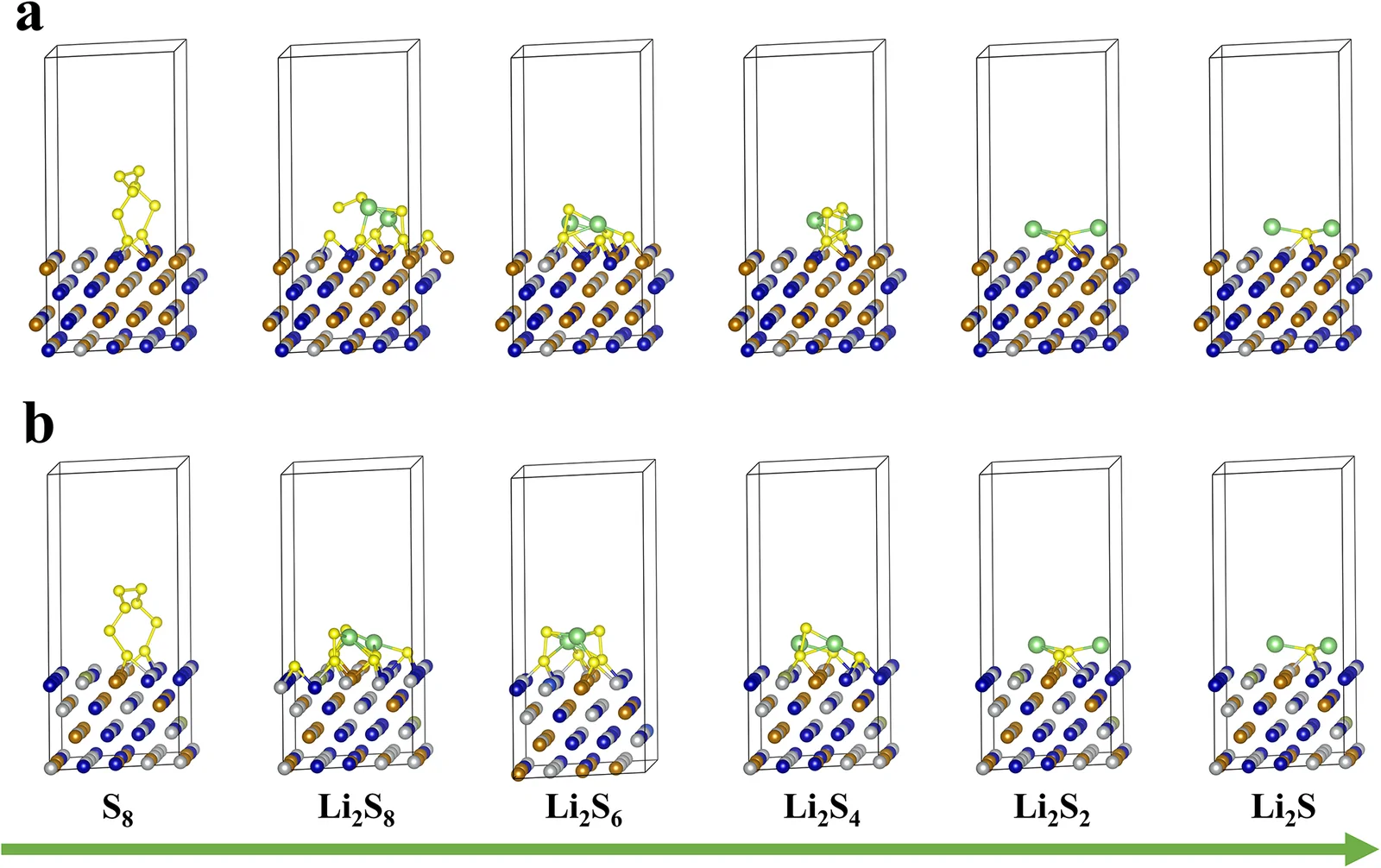
Optimized adsorption configurations of sulfur species on (a) FeCoNi and (b) FeCoNiCu MEA surfaces along the sulfur reduction pathway.

The optimized adsorption configurations of representative sulfur species on FeCoNi and FeCoNiCu surfaces are compared in Fig. 3. Both surfaces can accommodate the structural evolution from ring-like S_8_ and long-chain Li_2_S_8_/Li_2_S_6_ to shorter-chain Li_2_S_4_/Li_2_S_2_ and Li_2_S. For the FeCoNiCu surface, Cu incorporation increases the diversity of local coordination environments, allowing Li and S atoms to interact with mixed Fe/Co/Ni/Cu ensembles. Such a multimetallic interaction mode is beneficial for Li–S chemistry because soluble LiPSs require sufficient anchoring to suppress outward diffusion, whereas subsequent conversion requires facile electron transfer and flexible bond rearrangement. The full adsorption geometries for the FeCoNi and FeCoNiCu surfaces are provided in Fig. S3 and S4.

The adsorption configurations also suggest that the Cu effect should be interpreted as a cooperative surface effect. Fe, Co and Ni can provide polar and redox-active interaction sites for sulfur species, whereas Cu modifies the local electronic distribution around these neighboring sites. Such a multimetallic ensemble is different from a single-metal catalyst: the adsorbed LiPSs molecule can simultaneously interact with several metal atoms, and the final binding strength depends on the collective balance among metal–sulfur affinity, Li coordination and interfacial charge redistribution.

The low-energy Li_2_S_4_ and Li_2_S_6_ geometries further show why a small total Cu charge on the clean slab can coexist with a large adsorption-induced average Cu response. The polysulfides bridge mixed-metal ensembles, and Cu atoms occur within the local coordination environment of adsorbate Li/S atoms together with neighboring Fe, Co and Ni atoms (Fig. S4). Cu is therefore positioned to polarize locally in response to adsorption without serving as the principal charge reservoir of the entire slab. This geometric proximity supports the interpretation of Cu as a responsive local relay within a cooperative adsorption site.

To quantify surface-adsorbate interactions, the adsorption energy (*E*_ads_) was calculated using the following equation:

1 *E*_ads_ = *E*_adsorbate/surface_ − *E*_surface_ − *E*_adsorbate_

where *E*_ads_ refers to the adsorption energy, *E*_adsorbate/surface_ is the total energy of the optimized adsorption system, *E*_surface_ is the energy of the clean catalyst surface, and *E*_adsorbate_ is the energy of the isolated sulfur species.

**Fig. 4:**
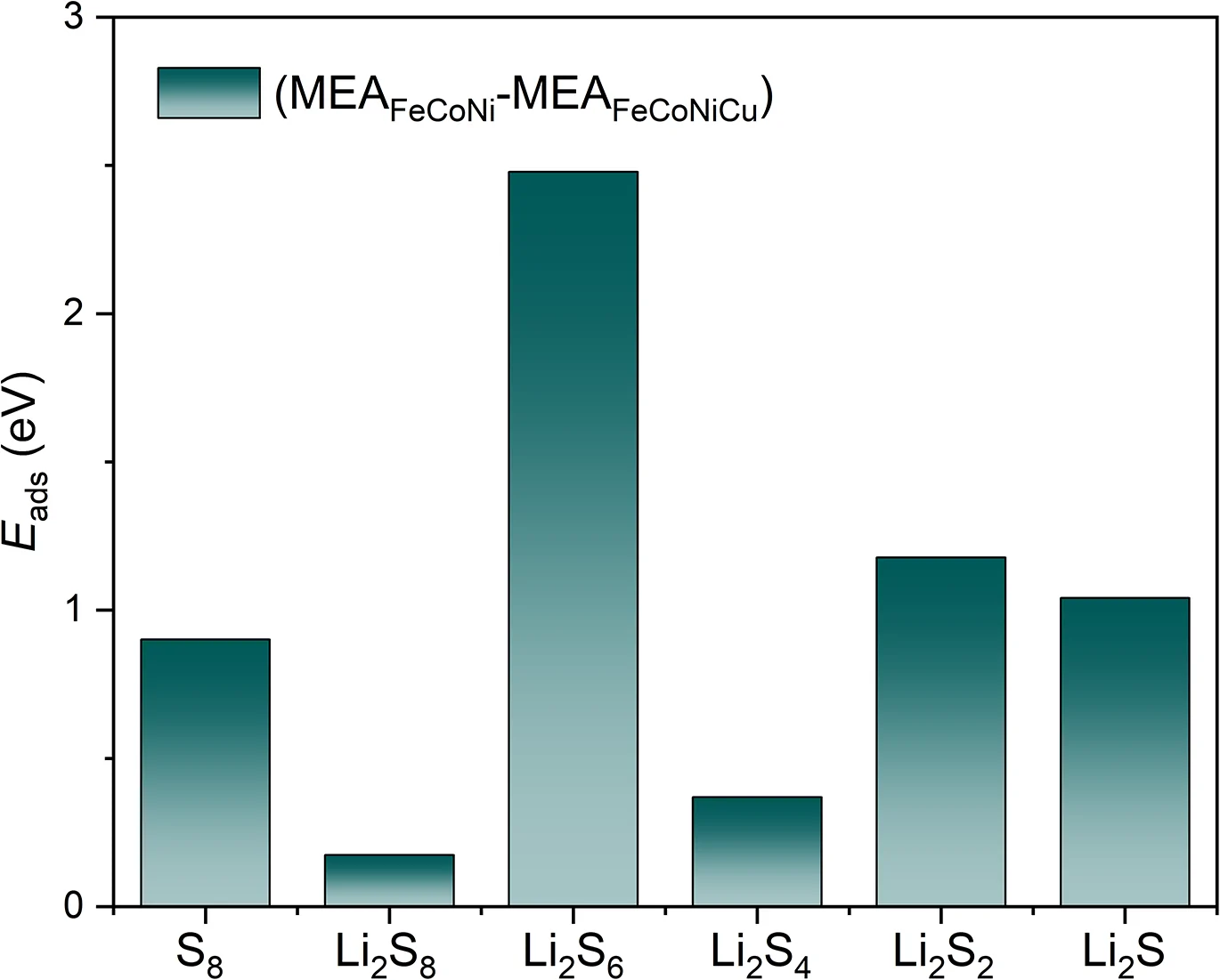
Adsorption-energy difference (Δ*E*_ads_ = *E*_ads_(FeCoNi) − *E*_ads_(FeCoNiCu)) between FeCoNi and FeCoNiCu MEA surfaces for representative sulfur species. Positive Δ*E*_ads_ values indicate more negative adsorption energies and therefore stronger adsorption on FeCoNiCu.

Based on this definition, the adsorption-energy difference is defined as Δ*E*_ads_ = *E*_ads_(FeCoNi) − *E*_ads_(FeCoNiCu) and summarized in Fig. 4. More negative *E*_ads_ values correspond to stronger adsorption; therefore, positive Δ*E*_ads_ values indicate more negative adsorption energies and stronger adsorption on FeCoNiCu. Among the studied species, Li_2_S_6_ exhibits the most pronounced adsorption enhancement after Cu incorporation, which is significant because soluble long-chain LiPSs are major contributors to the polysulfide shuttle effect. By contrast, the adsorption enhancement for Li_2_S_4_ is comparatively modest, implying that Cu incorporation does not simply strengthen every intermediate to the same extent.

This selective enhancement is important from the viewpoint of adsorption-conversion balance. If a catalyst only increases adsorption strength without considering conversion, overly stable surface species may slow the subsequent sulfur-reduction pathway. The present FeCoNiCu model instead shows stronger interaction with soluble LiPSs while maintaining a comparable free-energy profile for later reduction steps. Therefore, the computational result supports a nuanced conclusion: Cu incorporation modulates the adsorption-conversion balance of FeCoNi-based MEAs rather than merely producing the strongest possible binding.

### Sulfur-reduction thermodynamics on FeCoNi and FeCoNiCu surfaces

To further examine whether the improved adsorption remains compatible with sulfur conversion thermodynamics, the sulfur reduction process was expressed by the following elementary electrochemical steps:2a
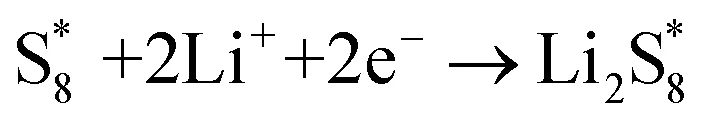
2b
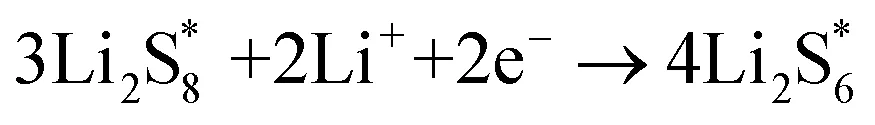
2c
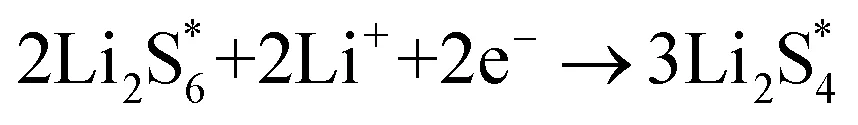
2d
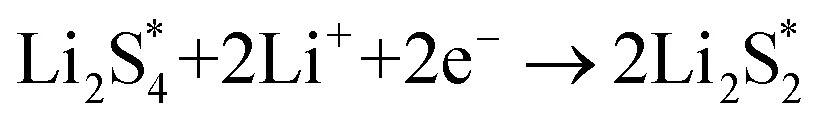
2e
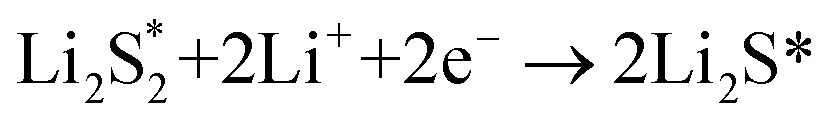
In the present FeCoNi/FeCoNiCu comparison, the main free-energy pathway focuses on the 
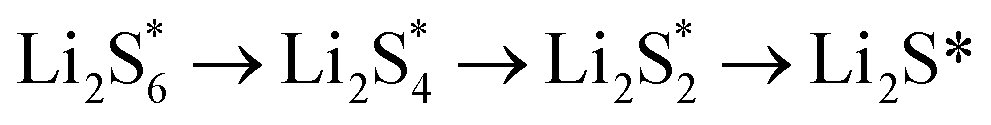
 segment, where * denotes the adsorbed state.

The Gibbs free-energy change for each sulfur-reduction step was calculated using the computational lithium electrode model under the zero-potential condition:^35^

3 Δ*G* = Δ*E*_DFT_ + ΔZPE − *T*Δ*S*

where Δ*E*_DFT_ is the electronic-energy difference between product intermediates and reactants obtained from DFT calculations. The chemical potential of the Li^+^ + e^−^ pair was referenced to the total energy of bulk metallic lithium. ΔZPE, Δ*S* and *T* represent the zero-point-energy change, entropy change and absolute temperature (*T* = 298.15 K), respectively.

**Fig. 5:**
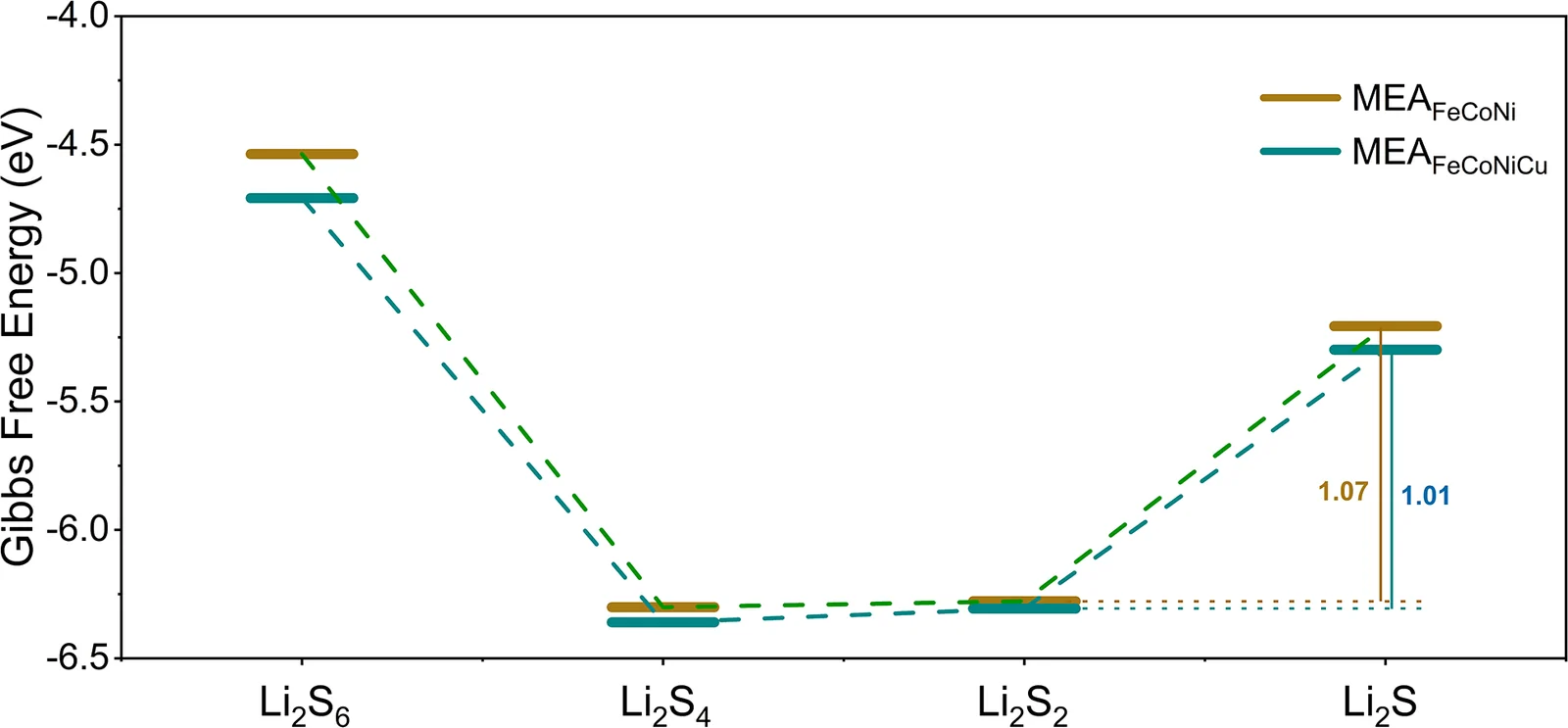
Gibbs free-energy profiles for sulfur reduction on FeCoNi and FeCoNiCu MEA surfaces under the zero-potential condition. The 
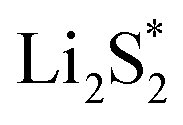
 to Li_2_S* conversion is identified as the most energetically demanding step.

As shown in Fig. 5, both surfaces exhibit comparable sulfur-reduction pathways, with the 
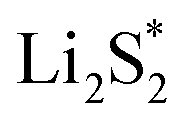
 to Li_2_S* conversion identified as the most energetically demanding step. After Cu incorporation, this limiting free-energy change decreases from 1.07 eV on FeCoNi to 1.01 eV on FeCoNiCu.

Although the decrease is moderate, it is mechanistically meaningful when combined with the adsorption results. The FeCoNiCu surface simultaneously enhances long-chain LiPSs anchoring and slightly lowers the limiting free-energy change associated with final solid-product formation. This dual behavior is preferable to stronger adsorption achieved at the expense of more difficult conversion. Thus, the DFT results suggest that Cu can improve the high-rate tolerance of FeCoNi-based catalytic interlayers, where rapid LiPSs capture and continuous electron-transfer-driven conversion are both required.

### Electrochemical feasibility of the DFT-motivated FeCoNiCu@CP interlayer

Motivated by the convergent computational evidence, a FeCoNiCu-containing catalytic interlayer was prepared on conductive carbon paper through precursor impregnation and ultrafast heating. This concise experiment provides a practical proof of concept: it verifies that the FeCoNiCu composition analyzed computationally can be realized as a working cathode-side interlayer under electrochemical conditions.^36–39^

**Fig. 6:**
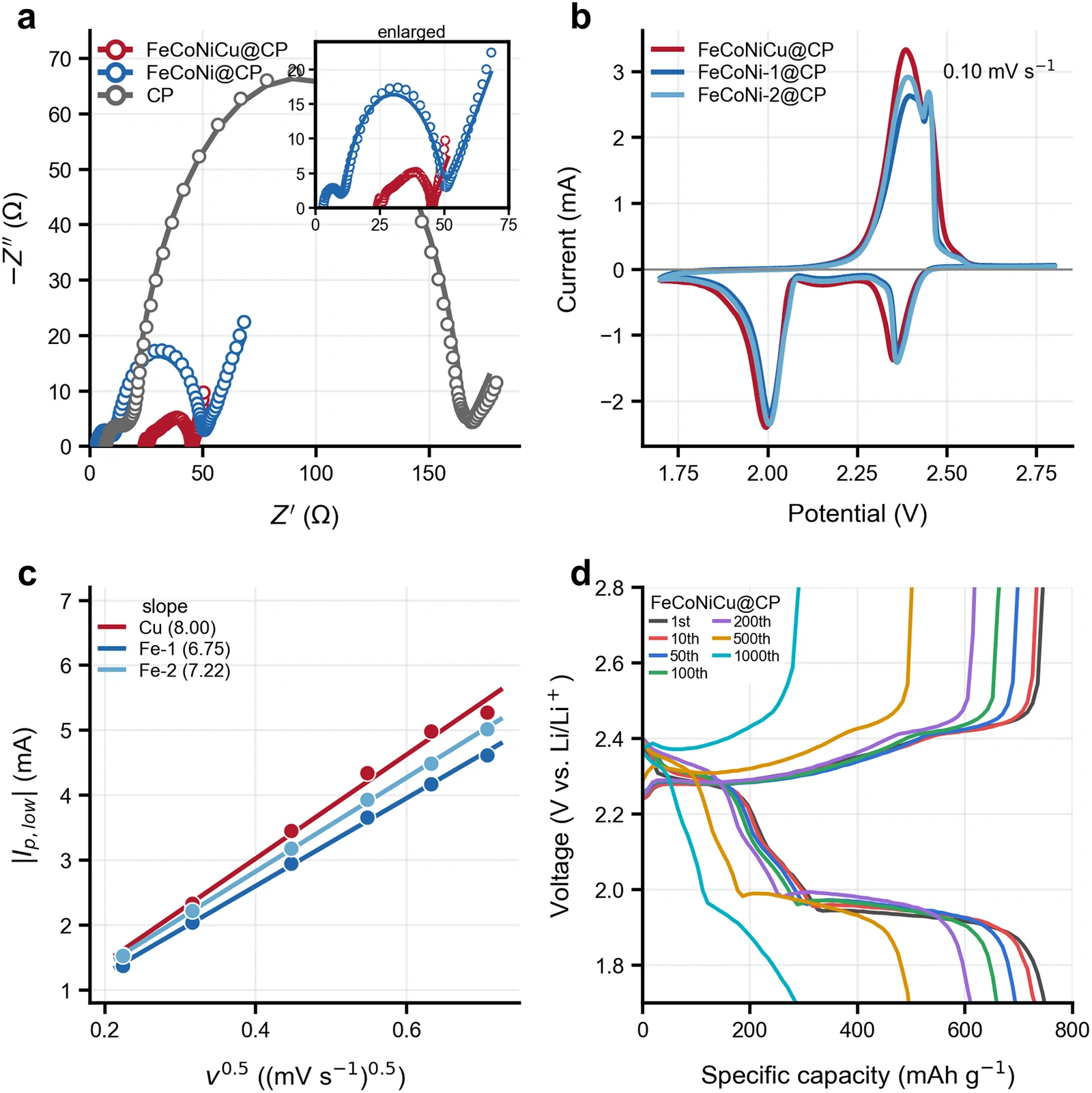
Matched-control electrochemical evaluation of the FeCoNiCu@CP catalytic interlayer. (a) Measured and fitted Nyquist plots of Li_2_S_6_ symmetric cells using FeCoNiCu@CP, FeCoNi@CP and pristine CP; the inset enlarges the catalyst-response region. (b) Second-cycle CV profiles of FeCoNiCu@CP and two FeCoNi@CP replicates at 0.10 mV s^−1^. (c) Low-potential cathodic peak-current magnitude *versus* the square root of scan rate; legend values are fitted slopes. (d) Representative selected-cycle galvanostatic charge–discharge profiles measured for FeCoNiCu@CP; no FeCoNi@CP profile is inferred where a matched raw profile is unavailable.

The electrochemical measurements provide a controlled validation layer for the computation-centered mechanism. The reviewer-requested three-sample Li_2_S_6_ symmetric-cell EIS and matched CV analysis are integrated directly into Fig. 6a–c, while panel d retains the measured selected-cycle FeCoNiCu@CP galvanostatic profiles. To keep the main figure focused, the raw matched 2C cycling trajectory and its unsmoothed early-cycle enlargement are provided in Fig. S5 and Table S1; the full extended FeCoNiCu@CP 2C trajectory remains in Fig. S6. Detailed EIS, preparation and CV values are reported in Tables S2–S4. These measurements test whether adding Cu under the same nominal preparation basis produces a consistent kinetic response; the adsorption, thermodynamic, DOS and Bader calculations provide the local atomistic interpretation.

Li_2_S_6_ symmetric-cell electrochemical impedance spectroscopy was used to probe interfacial polysulfide-conversion kinetics. In a symmetric cell, both electrodes are identical and the electrolyte contains soluble Li_2_S_6_; thus, the impedance response mainly reflects the interaction between the electrode surface and polysulfide redox species. The complete measured and fitted spectra show the smallest high-to-medium-frequency response for FeCoNiCu@CP, followed by FeCoNi@CP and pristine CP (Fig. 6a), consistent with progressively lower apparent charge-transfer resistance at the LiPSs-electrode interface.

The frequency-resolved spectra were fitted using the same modulus-weighted *R*_s_ − (R1‖CPE1) − (*R*_ct_‖CPE2) − W equivalent circuit. The fitted *R*_ct_ values are 10.74 Ω for FeCoNiCu@CP, 38.23 Ω for the newly measured FeCoNi@CP control and 144.89 Ω for pristine CP. The corresponding relative fit RMSE values are 1.67%, 4.48% and 3.42%, respectively. The ordering FeCoNiCu@CP < FeCoNi@CP < CP is consistent with an improved apparent Li_2_S_6_ interfacial charge-transfer response after Cu incorporation. Because the FeCoNi value derives from one spectrum and its fitted *R*_s_ differs appreciably from that of FeCoNiCu@CP, this result is treated as a same-protocol, within-cell comparison rather than a replicate-derived population statistic (Fig. 6a and Table S2).

Matched variable-scan-rate CV data were exported from VersaStudio and analyzed using the second complete cycle at 0.05–0.50 mV s^−1^ for FeCoNiCu@CP and two FeCoNi@CP replicates (Fig. 6b, c and Table S4). At 0.10 mV s^−1^, FeCoNiCu@CP gives anodic, high-potential cathodic and low-potential cathodic peak-current magnitudes of 3.290, 1.237 and 2.334 mA, compared with FeCoNi replicate means of 2.758, 1.129 and 2.131 mA. The corresponding high- and low-potential peak separations are 0.031 and 0.390 V for FeCoNiCu@CP and 0.033 and 0.401 V for the FeCoNi mean. Across all six scan rates, the low-potential cathodic *I*_p_ − *v*^1/2^ slope is 8.00 mA (mV s^−1^)^−1/2^ for FeCoNiCu@CP, compared with 6.75 and 7.22 for the two FeCoNi replicates, whereas the anodic and high-potential cathodic slopes are not universally larger after Cu incorporation. All linear fits give *R*^2^ = 0.991–1.000. Under equal-area, concentration and electron-number assumptions, the squared low-potential slope ratio relative to the FeCoNi replicate mean is 1.31. We therefore interpret the CV result as selective enhancement of the lower-potential conversion step, not as a universal improvement of every redox descriptor or as an absolute bulk Li^+^ diffusion coefficient.

The practical feasibility of the FeCoNiCu@CP catalytic interlayer was further evaluated by high-rate galvanostatic cycling against the nominally matched FeCoNi@CP control. Fig. S5 shows the raw cycle-summary trajectories from cycle 10 to 1000 and an enlarged view of cycles 10–150 without deleting or smoothing the transient short-capacity cycles in the FeCoNi@CP trace. To avoid overemphasizing activation behavior in the first few cycles, the 10th cycle was selected as the stabilized reference point. FeCoNiCu@CP provides 739.0 mA h g^−1^ at cycle 10 compared with 608.0 mA h g^−1^ for FeCoNi@CP, supporting a higher early utilization under the matched protocol. Fig. 6d separately shows the measured selected-cycle FeCoNiCu@CP galvanostatic profiles; a corresponding FeCoNi@CP profile series is not reconstructed in the absence of matched raw profile data.

Based on the stabilized 10th cycle, the FeCoNiCu@CP-containing cell retains 498.03 mA h g^−1^ after 500 cycles, corresponding to 67.40% capacity retention and an average capacity decay rate of 0.0665% per cycle. After 1000 cycles, the discharge capacity is 284.79 mA h g^−1^, corresponding to 38.54% retention and an average decay rate of 0.0621% per cycle relative to the 10th cycle. These values show clear capacity fading during prolonged high-rate operation; therefore, the result is best described as extended high-rate cycling tolerance rather than universally superior capacity retention. The corresponding full 2C cycling profile and data-treatment basis are provided in Fig. S6 and SI Note S4.

The matched 2C data provide the direct within-study comparison (Fig. S5 and Table S1). From cycle 10 to 1000, FeCoNiCu@CP changes from 739.0 to 284.8 mA h g^−1^, whereas FeCoNi@CP changes from 608.0 to 290.0 mA h g^−1^. The corresponding capacity-decay rates are 0.062% and 0.053% cycle^−1^, respectively. FeCoNiCu@CP therefore shows a clear early-capacity advantage (+21.5% at cycle 10 and +6.8% at cycle 100) and a smoother raw early-cycle response: the median absolute adjacent-cycle capacity change over cycles 10–100 is 0.19% for FeCoNiCu@CP *versus* 2.54% for the single FeCoNi@CP control. The unfiltered FeCoNi trace contains several transient short-capacity cycles that recover, so this variability comparison is descriptive rather than a replicate-derived statistic. The curves converge by cycle 1000. These results support improved early utilization and operational high-rate stability, not improved long-term retention, and also make the substantial fading limitation explicit.

Representative literature reports include Cu-free FeCoNi hosts, FeCoNiMnZn nano-alloy separator coatings, CoNiFePdV interlayers and PtCuFeCoNi cathode catalysts.^40^ These studies confirm that compositionally complex catalysts can support Li–S conversion, but differences in architecture, sulfur loading, electrolyte amount, catalyst placement and cycling protocol preclude a direct performance ranking and cannot independently isolate the role of Cu. The mechanistic conclusion is therefore based on the matched within-study FeCoNiCu@CP/FeCoNi@CP comparison rather than cross-study capacity values.

The capacity decrease from 738.97 mA h g^−1^ at cycle 10 to 284.79 mA h g^−1^ at cycle 1000 indicates that high-rate tolerance does not eliminate long-term degradation. Without post-mortem characterization, a unique failure mechanism cannot be assigned. Plausible contributions include continuing loss of soluble sulfur species, progressive accumulation of electronically insulating Li_2_S_2_/Li_2_S on the interlayer, electrolyte depletion and interfacial/contact degradation during repeated high-rate conversion. These mechanisms are presented as testable hypotheses rather than experimentally established causes. The result is therefore described as extended 2C operability, not superior long-term retention.

### Electronic-structure origin of Cu-mediated modulation

Although the adsorption-energy and free-energy results suggest that Cu incorporation improves the adsorption-conversion balance of FeCoNi-based MEA surfaces, the electronic origin of this modulation still requires further clarification. Therefore, density of states (DOS) and Bader charge analyses were further performed to identify how Cu affects the electronic structure and interfacial charge redistribution of the multimetallic surface. The DOS comparison among FeCoNi and FeCoNi-TM models was used to evaluate whether Cu provides favorable electronic-state availability near the Fermi level relative to other candidate components, rather than to simply claim Cu as an independently superior catalytic center. In parallel, element-resolved Bader charge analysis was employed to clarify the actual charge-transfer role of Cu after Li_2_S_4_/Li_2_S_6_ adsorption. This combined analysis allows the Cu effect to be discussed as a local electronic-modulation function within the FeCoNiCu ensemble.

**Fig. 7:**
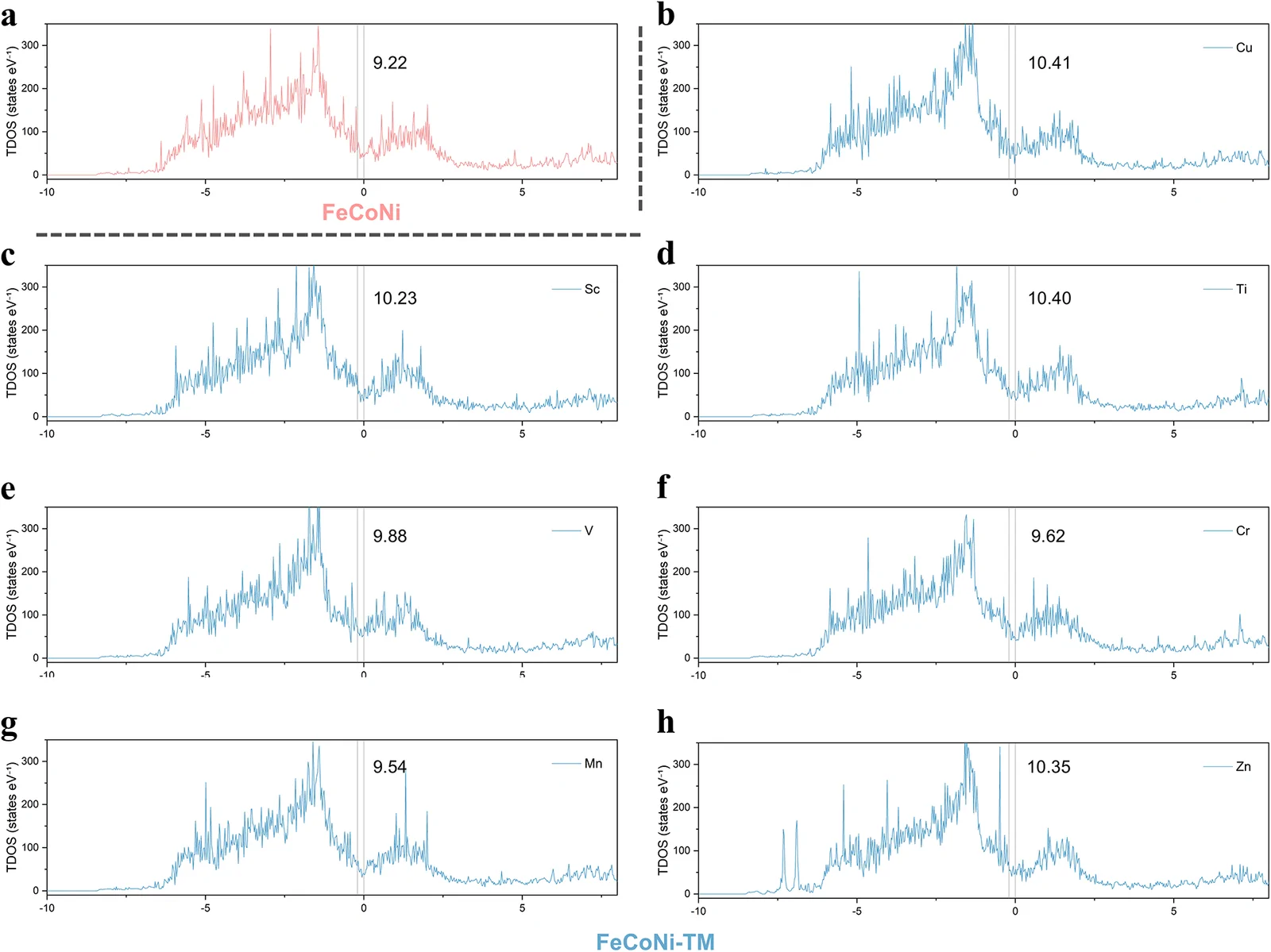
TDOS comparison of the FeCoNi and FeCoNi-TM (TM = Cu, Sc, Ti, V, Cr, Mn and Zn) models near the Fermi level. Panel (a) is FeCoNi, whereas panels (b)–(h) are the FeCoNi-TM models with Cu, Sc, Ti, V, Cr, Mn and Zn in the same order. The annotated value in each panel is the integrated TDOS from −0.2 to 0 eV and is used as a comparative descriptor of near-Fermi electronic-state availability.

The FeCoNi-TM TDOS comparison (TM = Sc, Ti, V, Cr, Mn, Cu and Zn) was not used as a post hoc ranking from which Cu was selected. Cu was the predefined mechanistic target because of its recurrent use as an additional component in the established Fe–Co–Ni–Cu multimetallic family, including the representative FeCoNiCu_0.5_ composition reported previously.^32^ The TDOS screen instead asks whether this empirical compositional choice has a plausible electronic basis. Fig. 7 shows that Cu-, Ti- and Zn-containing models all provide comparably high near-Fermi electronic-state availability under the reported analysis, indicating that Cu belongs to the favorable high-TDOS candidate group while also confirming that this descriptor is not sufficient for selecting a universally optimal fourth element. The manuscript therefore does not claim Cu is intrinsically superior to Ti or Zn; Cu is examined as a hypothesis-driven case study using the combined evidence of literature relevance, adsorption-conversion balance and adsorption-induced local charge responsiveness.

The newly extracted spin-resolved dopant d-band centers further show that similar near-Fermi TDOS integrals can arise from distinct orbital-energy distributions. Relative to *E*_F_ = 0 eV, the Ti d-band centers are +1.331/+0.762 eV (spin up/down), the Cu values are −2.551/−2.336 eV and the Zn values are −6.530/−6.622 eV. These data do not establish a universal activity ranking; instead, they reinforce that the TDOS integral alone cannot select Ti, Cu or Zn and that the Cu case must be interpreted together with its calculated adsorption-conversion balance and local Bader response (Table S5).

**Fig. 8:**
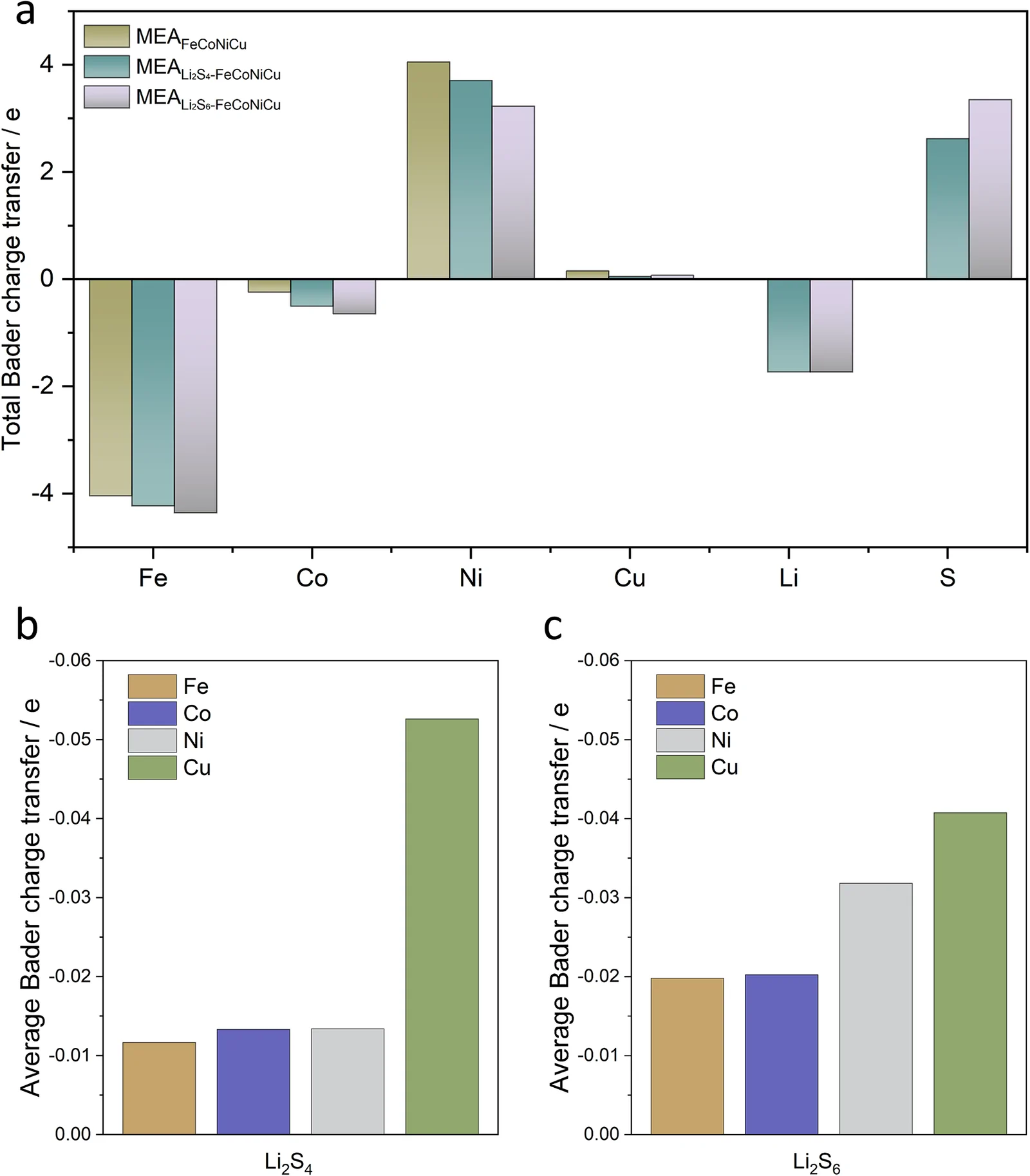
Bader charge-transfer analysis of the FeCoNiCu MEA system. (a) Total charge transfer for surface elements and adsorbed Li/S atoms before and after Li_2_S_4_/Li_2_S_6_ adsorption. Average charge transfer of surface Fe, Co, Ni and Cu atoms after adsorption of (b) Li_2_S_4_ and (c) Li_2_S_6_.

Bader charge analysis was then performed for the clean FeCoNiCu surface and for Li_2_S_4_/Li_2_S_6_ adsorption systems (Fig. 8 and S7–S10). On the clean surface, the exposed atoms are all metallic elements and the system lacks the more obvious adsorbate-surface electron exchange present after Li_2_S_4_/Li_2_S_6_ adsorption. The total clean-surface charge redistribution therefore remains dominated by Fe and Ni, whereas Co and Cu show smaller total changes; the single Cu atom consequently contributes only a small fraction of the clean-surface total charge response and behaves more like a local electronic regulator within the multimetallic lattice. This observation is consistent with a cooperative-catalysis picture in which the FeCoNi framework supplies the primary redox-active background and Cu adjusts the local electronic environment.

After Li_2_S_4_ adsorption, Cu displays the largest average adsorption-induced charge variation among the metal elements despite its small total contribution on the clean slab. In the optimized geometry, Li_2_S_4_ is coordinated by a mixed-metal environment in which Cu lies close to adsorbate Li/S atoms and adjacent Fe/Co/Ni sites. The large average variation therefore reflects a localized polarization response at the adsorption ensemble, whereas the slab-integrated charge remains dominated by the more numerous Fe/Ni reservoir sites.

A similar relationship is observed for Li_2_S_6_: the adsorbate spans a mixed Fe/Co/Ni/Cu environment and Cu again shows the largest average adsorption-induced response. This geometry–charge correlation is consistent with Cu acting as a local electronic relay that adjusts the charge distribution of neighboring active atoms during long-chain LiPS binding. It does not imply that Cu alone controls the entire redox pathway.

Combining total charge, average adsorption-induced variation and adsorption geometry yields a coherent division of roles: Fe and Ni provide the main charge reservoir, Co participates in cooperative redistribution, and Cu supplies a locally responsive degree of freedom within the adsorption ensemble. This is the central theoretical rationale for selecting Cu as a fourth component in the FeCoNi framework.

Taken together, these electronic-structure and charge-partitioning results support a mechanism in which Cu incorporation modulates, rather than independently dominates, the catalytic role of the FeCoNi framework. The FeCoNi surface provides the main multimetallic adsorption and redox-active background, whereas Cu creates additional local electronic heterogeneity and behaves as a responsive electronic regulator within the FeCoNiCu ensemble.

## Experimental

### Preparation of FeCoNiCu@CP catalytic interlayers

Single-metal precursor solutions were prepared by dissolving FeCl_3_·6H_2_O, CoCl_2_·6H_2_O, NiCl_2_·6H_2_O and CuCl_2_ in ethanol, respectively. According to the FeCoNiCu target composition, the corresponding precursor solutions were mixed and ultrasonicated to obtain a homogeneous multimetallic precursor solution. Commercial carbon paper was cleaned on both sides and punched into 19 mm discs. The precursor solution was drop-coated onto carbon paper placed on a 60 °C heating stage and kept for 20 min to allow solvent evaporation and precursor infiltration. The dried precursor-loaded carbon paper was then subjected to ultrafast Joule heating to promote multimetallic alloy formation on the conductive carbon framework. The obtained self-standing catalytic interlayer is denoted FeCoNiCu@CP.

For the matched FeCoNi@CP control, 1.333 mL each of 0.1 M Fe, Co and Ni precursor solutions were combined and diluted to 6.000 mL. The FeCoNiCu precursor used 1.000 mL each of 0.1 M Fe, Co, Ni and Cu precursor solutions and was likewise diluted to 6.000 mL. The hydrophilic TGP-H-060 carbon paper was air-plasma cleaned on both sides at 100 W for 15 min and punched into 19 mm discs. In both cases, 60 µL of precursor was drop-coated onto each disc on a 60 °C heating stage and held for 20 min, corresponding to the same nominal total-metal input of 4.00 µmol per disc. Joule heating was then applied identically at 30 V and 150 A for a single 50 ms pulse with the maximum temperature set to 1000 °C. Because an independent ICP or gravimetric determination is not available for every interlayer disc, the catalyst comparison is described as matched nominal loading rather than proven identical deposited mass. For the 2C full cells, the LANDdt sulfur-mass entries were 1.580 mg for FeCoNiCu@CP and 1.500 mg for FeCoNi@CP, corresponding to 1.20 and 1.14 mg cm^−2^ over a 1.32 cm^2^ cathode area; both fall within the 1.0–1.2 mg cm^−2^ preparation range and each capacity was normalized to its own recorded sulfur mass.

### Preparation of sulfur cathodes

Nano sulfur and Super P carbon were mixed at a mass ratio of 4 : 1 and ground for 20 min. The mixture was sealed and heated at 155 °C for 12 h to obtain S/Super P through melt diffusion. The cathode slurry was prepared by mixing S/Super P, carbon nanotubes and LA133 binder at a mass ratio of 7 : 2 : 1. The slurry was coated onto Al foil and dried at 55 °C in a vacuum oven for 8 h. Typical sulfur loading was controlled at 1.0–1.2 mg cm^−2^ unless otherwise specified.

### Cell assembly and electrochemical measurements

CR2032 coin cells were assembled in an Ar-filled glovebox using lithium metal foil as the anode, a polypropylene separator and a carbon-paper-based catalytic interlayer placed on the cathode side. The electrolyte consisted of 0.5 M LiTFSI and 0.5 M LiNO_3_ dissolved in DOL/DME (1 : 1 by volume). Galvanostatic charge–discharge tests were conducted within 1.7–2.8 V *versus* Li/Li^+^ on battery testing systems. High-rate cycling of FeCoNiCu@CP-containing cells was evaluated at 2C, and the 10th cycle was used as the stabilized reference point for capacity-retention and average-decay calculations. Representative galvanostatic charge–discharge profiles were extracted from the same 1000-cycle dataset.

### Li_2_S_6_ symmetric cells

Li_2_S_6_ symmetric cells were assembled using two identical carbon-paper-based electrodes. Li_2_S_6_ solution was prepared by reacting Li_2_S and sulfur at a stoichiometric molar ratio of 1 : 5 in LS-072 electrolyte under continuous stirring at 60 °C for 12 h. For each symmetric cell, 25 µL of 0.2 M Li_2_S_6_ solution was injected into each side. CV tests were performed within −1.0 to 1.0 V, and EIS was collected from 100 kHz to 0.01 Hz with an amplitude of 5 mV.

### DFT calculations

All density functional theory calculations were performed using the Vienna *Ab initio* Simulation Package.^41^ Core electrons were described by projector-augmented-wave potentials,^42^ and the spin-polarized Perdew–Burke–Ernzerhof functional within the generalized gradient approximation was adopted for exchange–correlation interactions.^43^ The plane-wave cutoff energy was set to 500 eV. The selected bulk models were cleaved along the (111) direction to construct four-layer slab models with a 15 Å vacuum layer along the surface-normal direction; the bottom two layers were fixed, whereas the top two layers and all adsorbates were fully relaxed. A 3 × 3 × 1 Monkhorst–Pack *k*-point mesh was used for slab optimization, a Gamma-only mesh was used for adsorbed polysulfide relaxation, and a 4 × 4 × 1 mesh was used for electronic-structure calculations.^44^ The convergence thresholds for total energy and atomic force were 10^−5^ eV and 0.02 eV Å^−1^, respectively. The Dudarev DFT + U formalism was applied to selected 3d transition-metal elements,^45^ with effective *U* values of 3.0 eV for Fe, 3.0 eV for Co and 2.5 eV for Ni; Cu was treated without an additional *U* correction. For the TDOS screening, electronic states were referenced to the Fermi level (*E*_F_ = 0 eV), and the near-Fermi TDOS integral was evaluated over the −0.2 to 0 eV window for comparison among FeCoNi and FeCoNi-TM models.

For each sulfur species, several representative initial adsorption configurations were tested when geometrically meaningful, and the optimized low-energy adsorption structures were used for the main-text comparison. The same computational settings were applied to FeCoNi and FeCoNiCu so that adsorption-energy, free-energy and charge-transfer differences could be assigned to Cu incorporation rather than to inconsistent numerical treatment. Bader charge analysis was carried out using a grid-based charge-partitioning algorithm^46,47^ to quantify interfacial electron redistribution before and after LiPSs adsorption.

Adsorption energies and Gibbs free-energy changes were evaluated according to the equations introduced in the main text. The values discussed in the free-energy profile are thermodynamic free-energy changes rather than kinetic transition-state quantities.

## Conclusions

In summary, by establishing a controlled FeCoNi/FeCoNiCu DFT comparison and evaluating the practical behavior of the DFT-motivated FeCoNiCu@CP catalytic interlayer, this work clarifies the plausible component-level regulatory role of Cu in FeCoNi-based multimetallic Li–S catalysis. DFT calculations show that Cu incorporation strengthens LiPS adsorption, especially for soluble Li_2_S_6_, increases near-Fermi electronic-state availability in the TDOS comparison, and decreases the limiting 
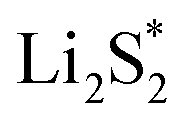
 to Li_2_S* free-energy change from 1.07 to 1.01 eV. Guided by this theoretical prediction, FeCoNiCu@CP was prepared and evaluated as a cathode-side catalytic interlayer. Li_2_S_6_ symmetric-cell EIS shows a smaller apparent charge-transfer semicircle than pristine FeCoNiCu@CP, suggesting facilitated interfacial charge transfer in a polysulfide-containing environment. At 2C, the FeCoNiCu@CP-containing cell delivers 738.97 mA h g^−1^ at the stabilized 10th cycle, retains 498.03 mA h g^−1^ after 500 cycles and 284.79 mA h g^−^1 after 1000 cycles.

Bader charge analysis further reveals that Fe/Ni provide the main charge-transfer reservoir, Co assists Fe-related charge donation and Cu acts as a highly responsive local electronic regulator during Li_2_S_4_/Li_2_S_6_ adsorption. These findings suggest that Cu incorporation improves the adsorption-conversion balance and electronic responsiveness of FeCoNi-based MEA surfaces, while the FeCoNiCu@CP interlayer exhibits practical high-rate cycling tolerance consistent with this mechanism.

## Author contributions

Conceptualization, X. G., X. S. and Y. F.; methodology, X. G. and J. W.; investigation, X. G. and J. W.; formal analysis, X. G.; software, X. G.; data curation, X. G.; writing – original draft preparation, X. G.; writing – review and editing, X. G., J. W., X. S., J. S. and Y. F.; supervision, X. S., J. S. and Y. F.; funding acquisition, X. S. and Y. F. All authors have read and agreed to the published version of the manuscript.

## Conflicts of interest

There are no conflicts to declare.

## Supplementary Material

RA-OLF-D6RA06165F-s001

## Data Availability

The data supporting this article, including experimental results, electrochemical data, DFT calculation results, optimized adsorption structures, DOS analysis, Bader charge analysis, and key quantitative conclusions, have been included in the article and its supplementary information (SI). No new software or code was developed for this study. Supplementary information: six supplementary notes, ten supplementary figures (Fig. S1–S10) and five supplementary tables (Tables S1–S5), covering the DFT model-composition and surface-selection audit and the adsorption/free-energy definitions (Notes S1–S2, Fig. S1–S4), Bader-charge and element-resolved d-band analyses for the clean and polysulfide-adsorbed slabs (Notes S3 and S6, Fig. S7–S10, Table S5), and the electrode preparation together with matched 2C cycling, symmetric-cell EIS and CV kinetic data for FeCoNiCu@CP and FeCoNi@CP (Notes S4–S5, Fig. S5–S6, Tables S1–S4). See DOI: https://doi.org/10.1039/d6ra06165f.
