# Supplementary material for: Theoretical insights into Cu-mediated electronic modulation in FeCoNi-based multimetallic catalysts for polysulfide conversion
Source: RSC Adv. 2026 Sep 18;16(44):55790–9. doi: 10.1039/d6ra06165f (PMC13588122; doi:10.1039/d6ra06165f)
Supplement: Supplementary file 1 [file RA-016-D6RA06165F-s001.pdf]

Supplementary Information (SI) for RSC Advances.
This journal is © The Royal Society of Chemistry 2026

Supporting Information

Theoretical Insights into Cu-Mediated Electronic Modulation in FeCoNi-Based Multimetallic Catalysts for Polysulfide Conversion

Xin Guo[a], Jianshuang Wei[d], Xiuju Song[c], Jingyu Sun[d], and Yixiong Feng*[a,b,c]

[a] State Key Laboratory of Public Big Data, Guizhou University, Guiyang 550025, Guizhou Province, China.

[b] College of Mechanical Engineering, Guizhou University, Guiyang 550025, Guizhou Province, China.

[c] State Key Laboratory of Fluid Power & Mechatronic Systems, Zhejiang University, Hangzhou 310058, China.

[d] College of Energy, Soochow Institute for Energy and Materials Innovations, Key Laboratory of Advanced Carbon Materials and Wearable Energy Technologies of Jiangsu Province, Soochow University, Suzhou, China.

* Corresponding author. E-mail: fyxtv@zju.edu.cn

Supplementary Notes

Supplementary Note S1. Model-composition and surface-selection boundary. The 20 folders labeled FeCoNi all have the fixed $Fe_{10}Co_{11}Ni_{11}$ composition, whereas all 20 Cu-containing folders have the fixed $Fe_8Co_{10}Ni_{13}Cu_1$ composition. Every OUTCAR reached the required electronic/ionic accuracy. Because high-/medium-entropy alloys are intrinsically disordered, these finite DFT cells are used to sample possible local atomic arrangements rather than to reproduce the full configurational ensemble. Configuration 5 has the lowest raw total energy within the fixed $Fe_{10}Co_{11}Ni_{11}$ set, and configuration 5 is also the lowest within the fixed $Fe_8Co_{10}Ni_{13}Cu_1$ group; raw energies were not ranked across Cu-free and Cu-containing stoichiometries. The Cu-containing cell is used as a dilute single-Cu perturbation model and is not described as an equimolar experimental replica or as a statistical ensemble average. The common (111) orientation is retained as a literature-supported representative surface based on exposed (111) fringes in $FeCoNiCu_{0.5}$ and the use of (111) as a representative close-packed facet in related FCC high-entropy-alloy calculations (refs. 32 and 34 in the main text); no claim of unique thermodynamic dominance is made for the exact present composition.

Supplementary Note S2. Adsorption-energy and free-energy definitions. Sulfur species including S_8, Li_2S_8, Li_2S_6, Li_2S_4, Li_2S_2 and Li_2S were placed on representative surface environments and relaxed. Adsorption energy was calculated as $E_{ads} = E_{adsorbate/surface} - E_{surface} - E_{adsorbate}$. In the main text, adsorption enhancement is plotted as E_{ads}(FeCoNi) − E_{ads}(FeCoNiCu), where positive values indicate stronger adsorption on FeCoNiCu. Free-energy changes were evaluated using the computational lithium electrode model under the zero-potential condition.

Supplementary Note S3. Bader charge and adsorption geometry. Total charge changes identify the principal charge reservoir of the clean FeCoNiCu slab, whereas average adsorption-induced changes

quantify the local response of each element. Because the clean surface consists only of metallic elements and lacks the stronger adsorbate–surface electron exchange present in Li_2S_4/Li_2S_6 adsorption models, the total clean-surface charge contribution associated with Cu is comparatively small. In the optimized Li_2S_4 and Li_2S_6 structures, the adsorbates bridge mixed Fe/Co/Ni/Cu environments and Cu occurs within the local coordination region of adsorbate Li/S atoms. The comparatively large average Cu variation is therefore interpreted as localized polarization within a cooperative adsorption ensemble, not as evidence that Cu dominates the total slab charge transfer.

Supplementary Note S4. FeCoNiCu@CP preparation and electrochemical data treatment. FeCoNiCu@CP was prepared using a carbon-paper precursor-impregnation/ultrafast-heating protocol adapted to the Fe–Co–Ni–Cu composition. For long-term 2C cycling, the stabilized 10th cycle was used as the reference point to avoid overinterpreting early activation. Capacity retention was calculated as CN/C10 × 100%, and the average decay rate was calculated as (1 - CN/C10)/(N - 10) × 100% per cycle.

Supplementary Note S5. Equivalent-circuit analysis of Li_2S_6 symmetric-cell EIS. The complete 100 kHz–0.01 Hz spectra were fitted using the same modulus-weighted R_s–(R_1||CPE_1)–(R_{ct}||CPE_2)–W circuit. The fitted R_{ct} values are 10.74 Ω for FeCoNiCu@CP, 38.23 Ω for FeCoNi@CP and 144.89 Ω for pristine CP, with relative RMSE values of 1.67%, 4.48% and 3.42%, respectively. The FeCoNi spectrum was collected using the same CHI760E frequency range and 5 mV amplitude as the FeCoNiCu spectrum, and both catalyst electrodes used the same nominal total-metal input and documented downstream fabrication procedure. Because the FeCoNi result is based on one spectrum and its fitted R_s differs from that of FeCoNiCu@CP, it is interpreted as a same-protocol apparent R_{ct} comparison rather than a replicate-derived population statistic.

Supplementary Note S6. Dopant-resolved d-band centers. Element-resolved PDOS outputs for Sc, Ti, V, Cr, Mn, Cu and Zn were analyzed over −14 to 8 eV relative to E_F = 0 eV. The spin-resolved d-band centers are reported in Table S5. These quantities are used to show that similar near-Fermi TDOS integrals do not imply identical orbital distributions; they are not interpreted as a stand-alone catalytic activity ranking.

Supplementary Figures

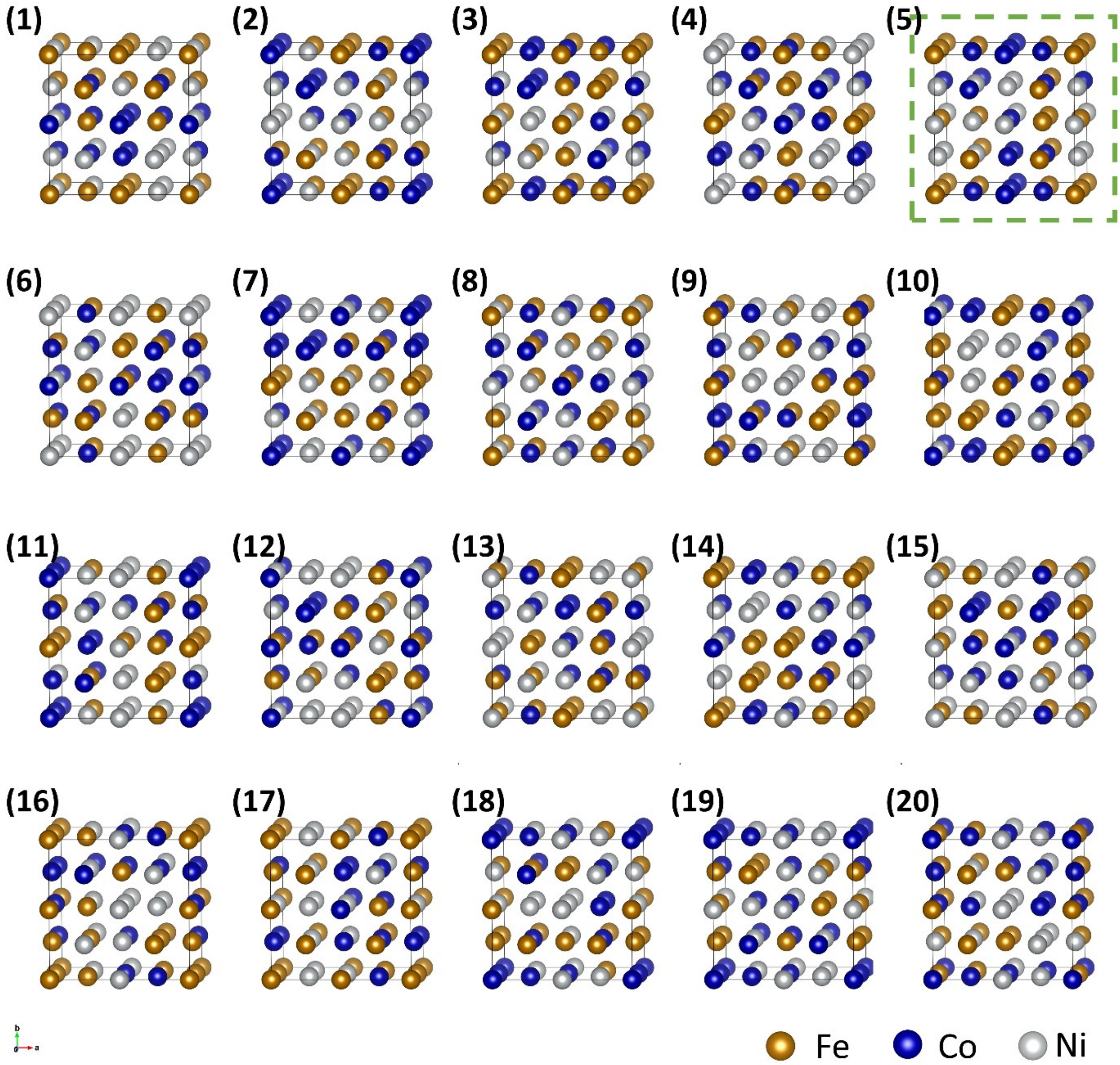

Figure S1. Source-audit view of the 20 folders labeled FeCoNi. All 20 files have the $Fe_{10}Co_{11}Ni_{11}$ composition. The green-framed structure (5) is the lowest-energy representative within this fixed-composition set, but it is not interpreted as the statistical average of the disordered alloy.

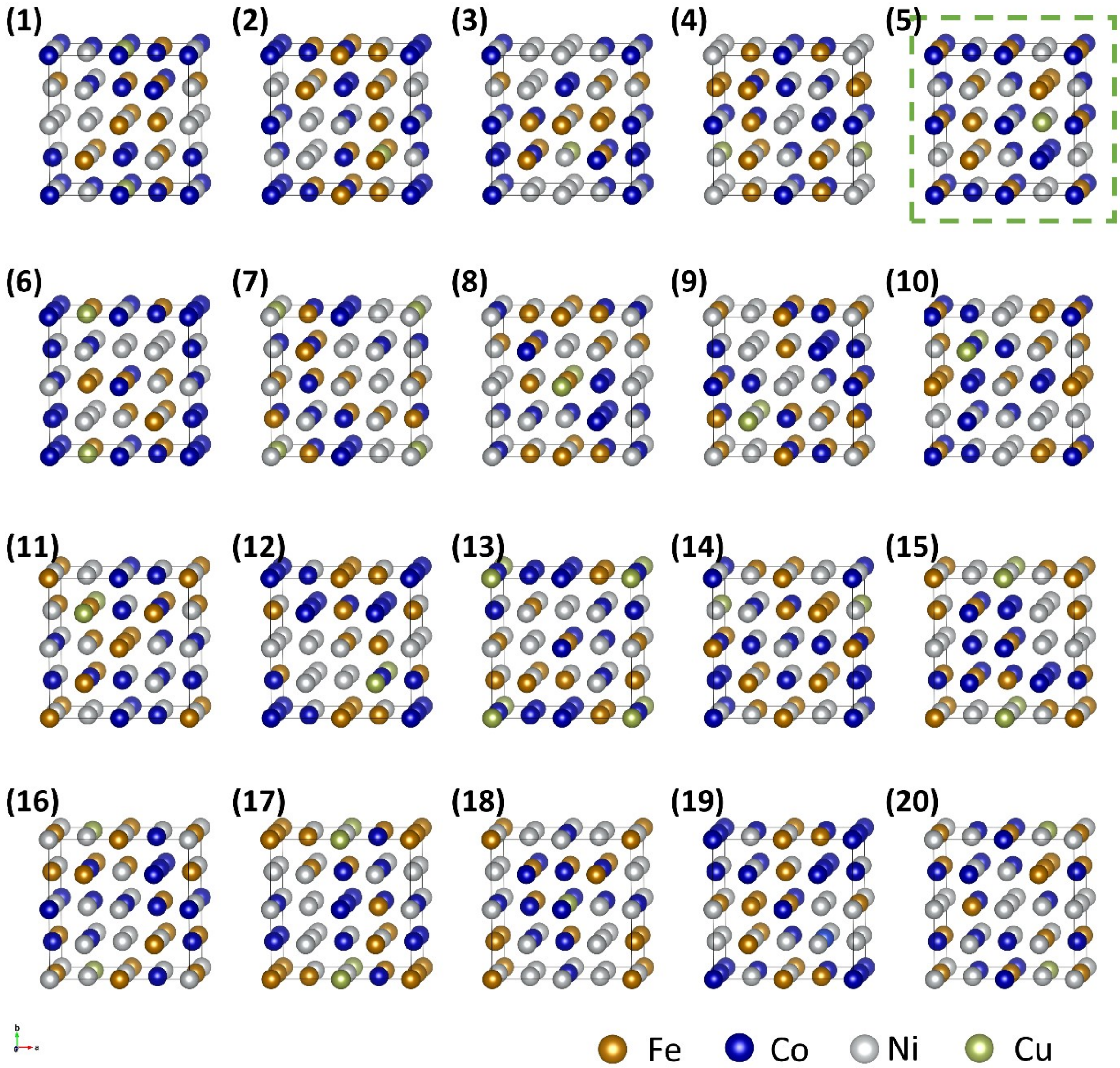

Figure S2. Source-audit view of the 20 folders labeled FeCoNiCu. All files have the dilute-Cu composition $Fe_8Co_{10}Ni_{13}Cu_1$, and structure (5) has the lowest raw total energy within this fixed composition. The set does not represent nominally equimolar FeCoNiCu.

a

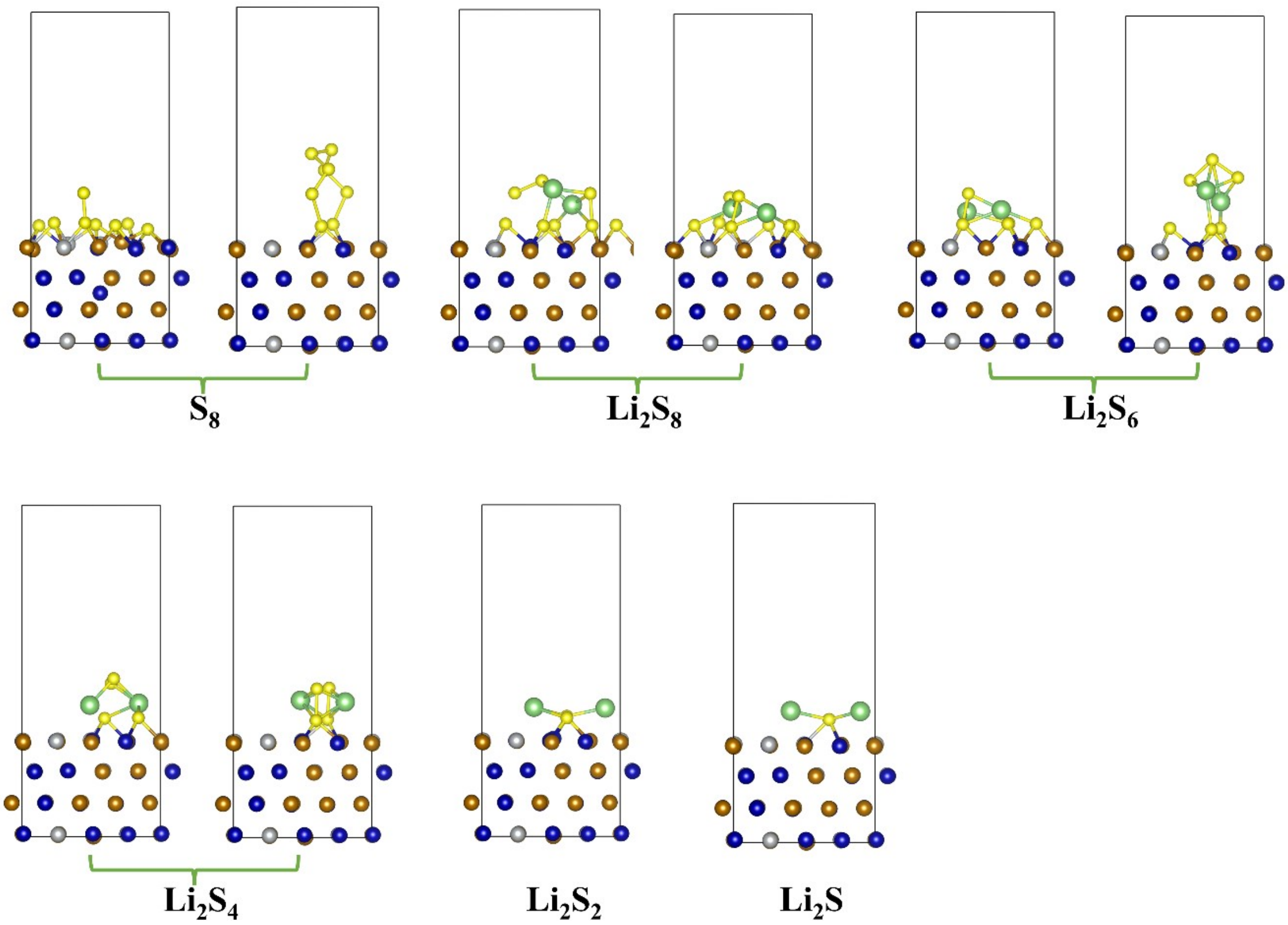

Figure S3. Optimized adsorption configurations of S8, Li_2S_8, Li_2S_6, Li_2S_4, Li_2S_2 and Li_2S on the representative $Fe_{20}Co_{22}Ni_{22}$ (111) surface. This model is not interpreted as an ensemble average.

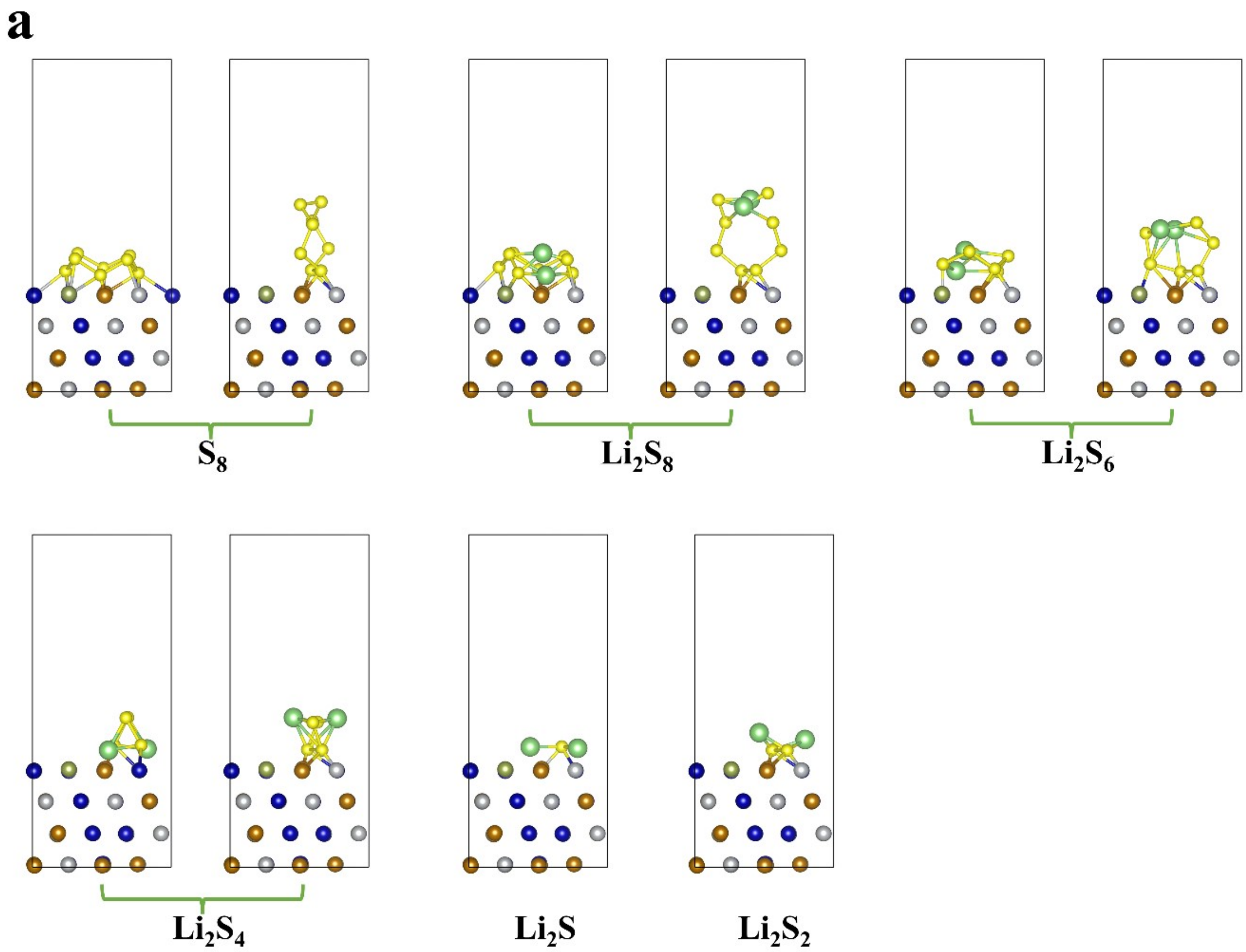

Figure S4. Optimized adsorption configurations of S8, Li_2S_8, Li_2S_6, Li_2S_4, Li_2S_2 and Li_2S on the representative dilute-Cu $Fe_{16}Co_{20}Ni_{26}Cu_2$ (111) slab derived from the $Fe_8Co_{10}Ni_{13}Cu_1$ bulk cell. The model probes a local Cu perturbation and is not an equimolar experimental replica.

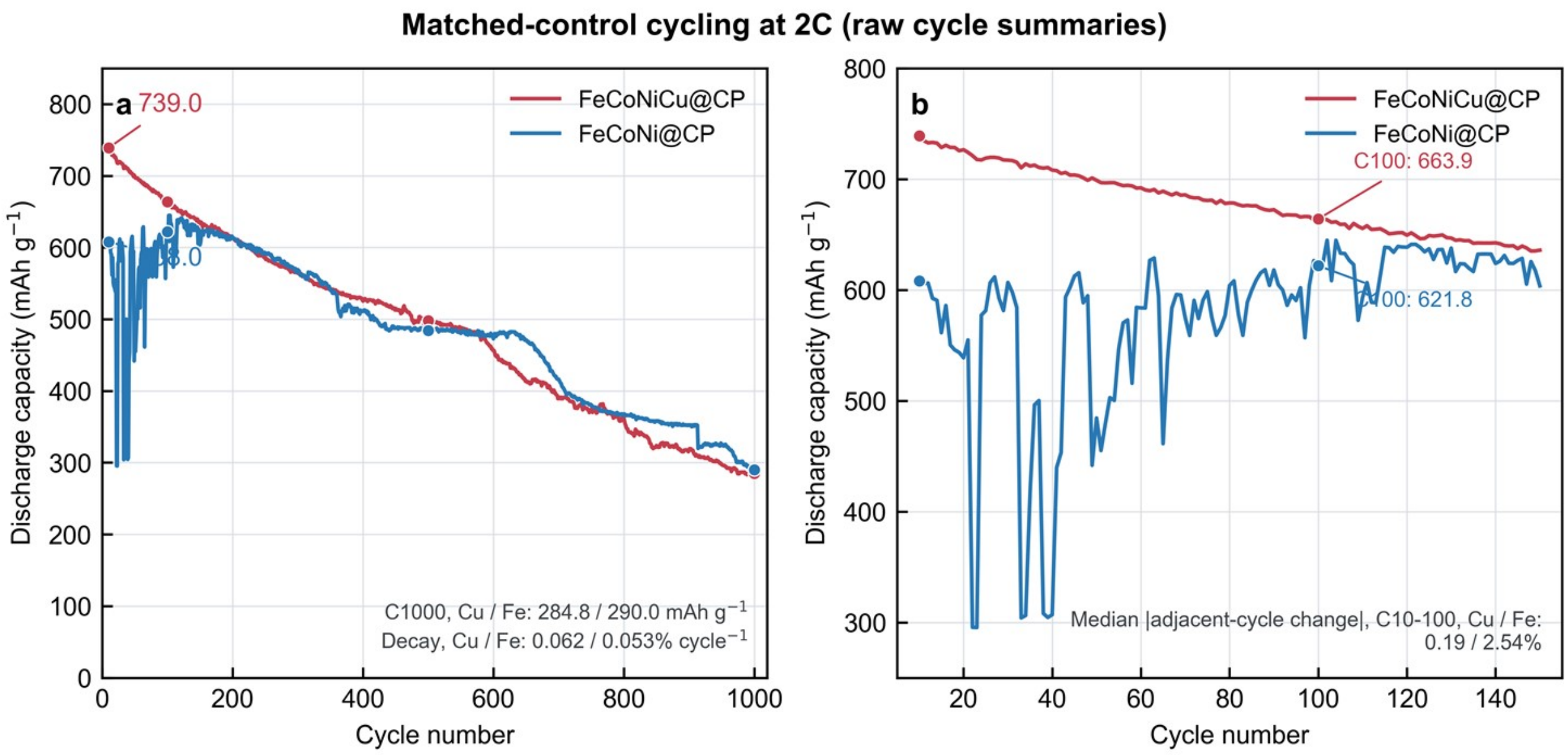

Figure S5. Raw, unsmoothed matched FeCoNiCu@CP and FeCoNi@CP cycling at 2C. (a) Sulfur-mass-normalized discharge capacity from cycle 10 to 1000. (b) Expanded view of cycles 10–150. The LANDdt sulfur-mass entries are 1.580 and 1.500 mg, respectively. FeCoNiCu@CP shows a higher and smoother early response; the FeCoNi@CP control contains several transient short-capacity cycles between cycles 22 and 65 that recover and have not been deleted or smoothed. The median absolute adjacent-cycle capacity changes over cycles 10–100 are 0.19% and 2.54%, respectively. This is a descriptive single-cell comparison, while the full trajectories converge by cycle 1000.

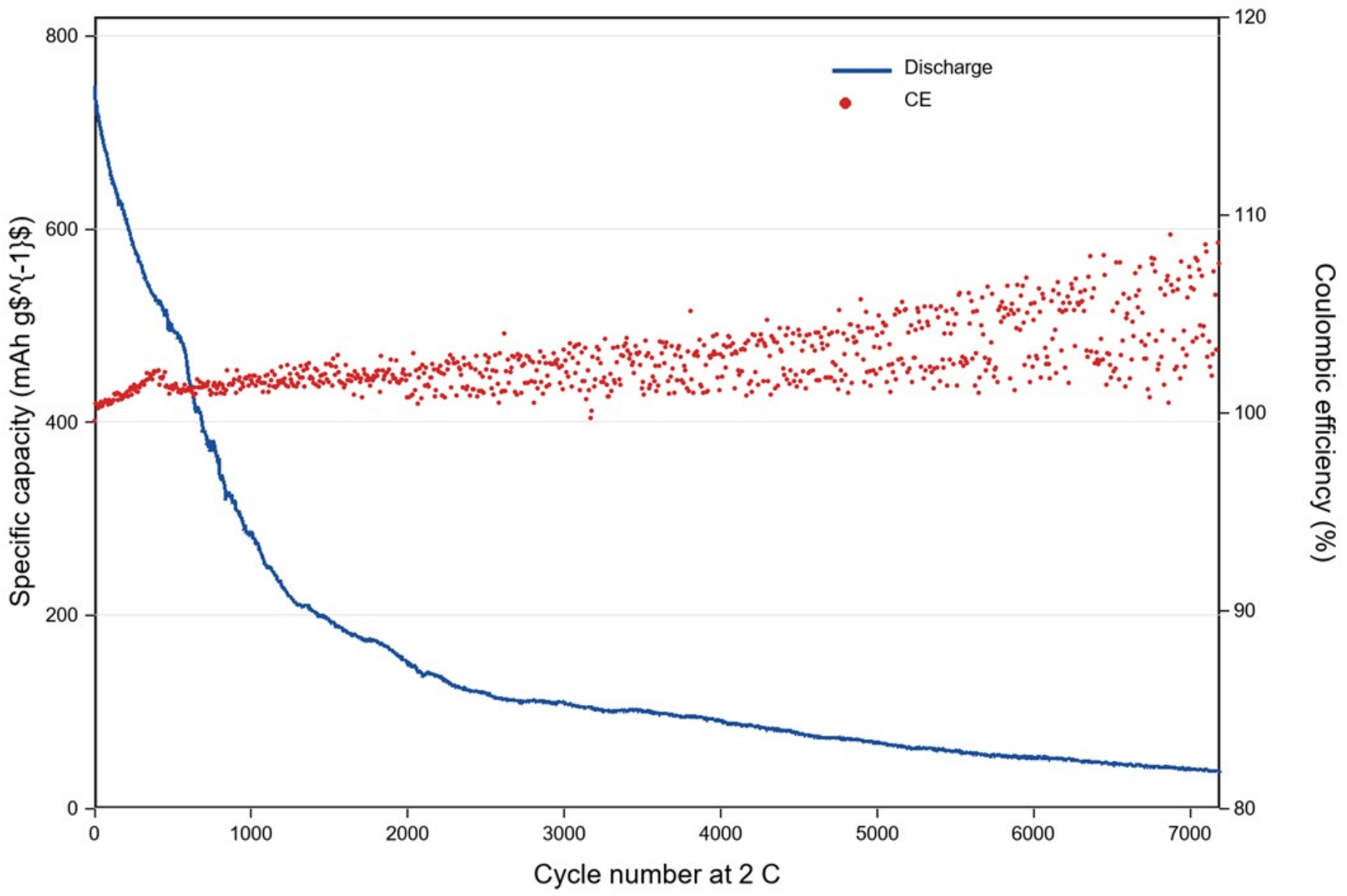

Figure S6. Full 2C cycling profile of the FeCoNiCu electrode. The main-text discussion uses the stabilized 10th cycle as the reference point for retention and average-decay calculations.

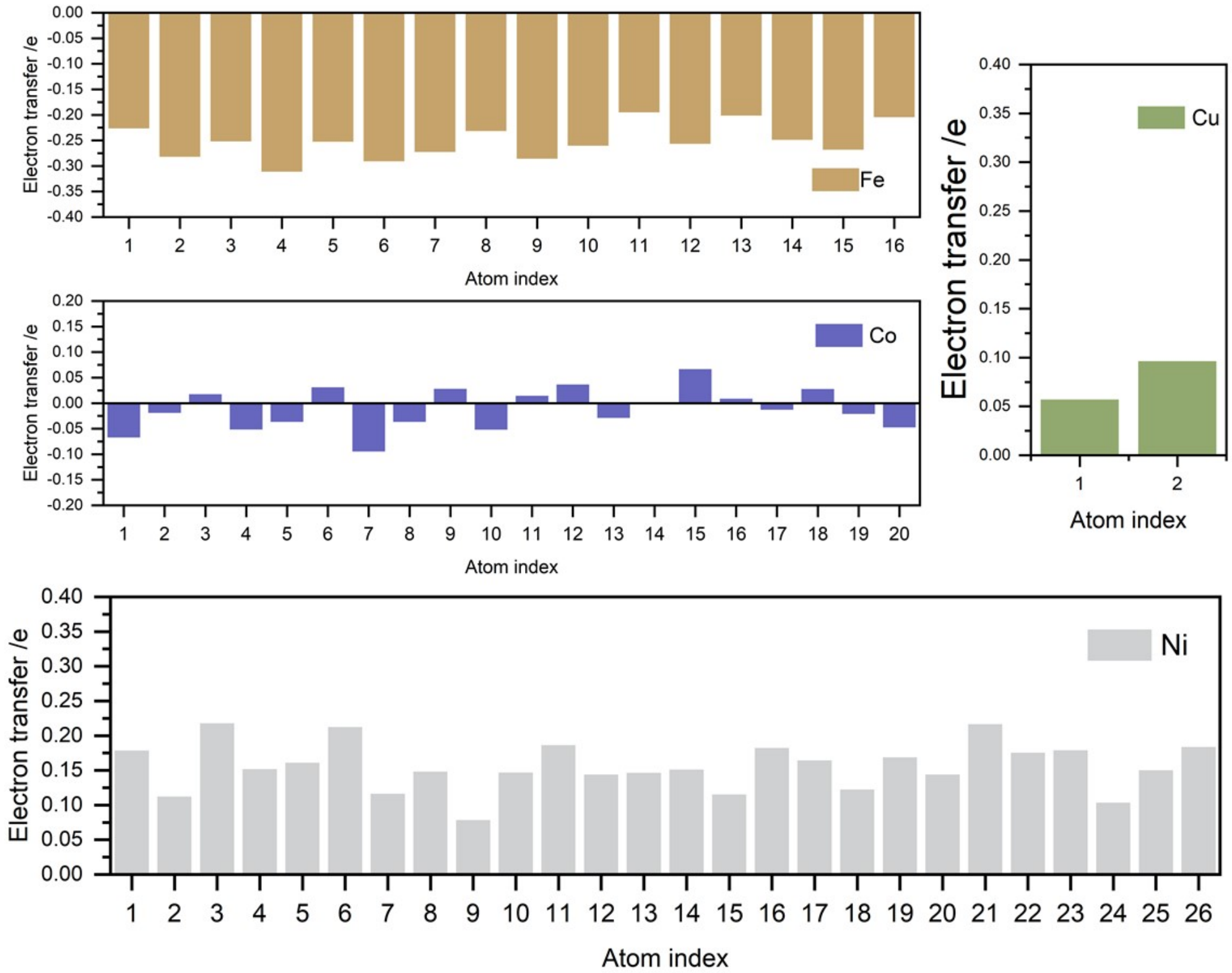

Figure S7. Bader charge analysis of surface atoms on the clean FeCoNiCu MEA surface before polysulfide adsorption, used to identify total charge redistribution among Fe, Co, Ni and Cu.

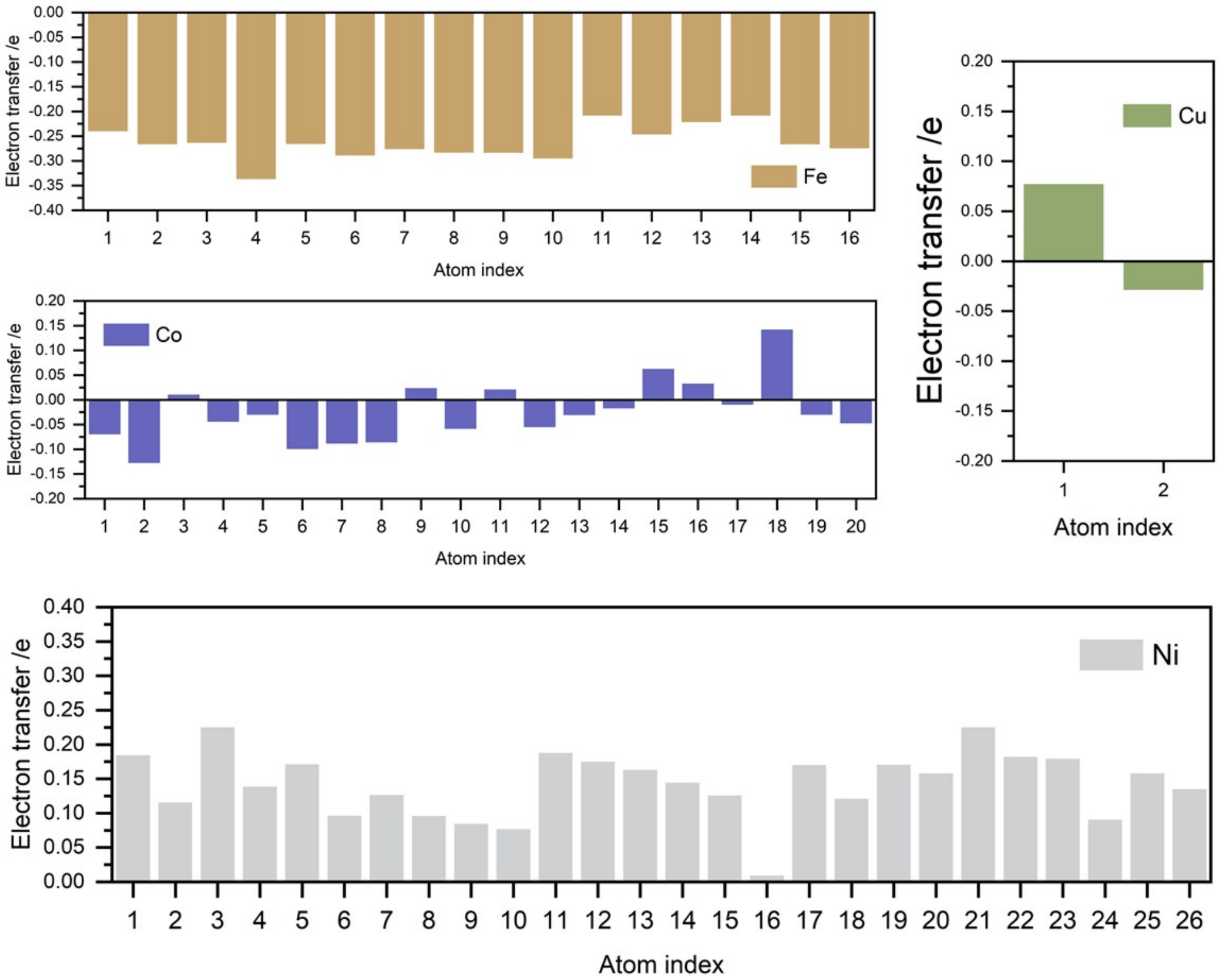

Figure S8. Bader charge analysis of the FeCoNiCu MEA system after Li_2S_4 adsorption, showing element-resolved charge response during short-chain polysulfide adsorption.

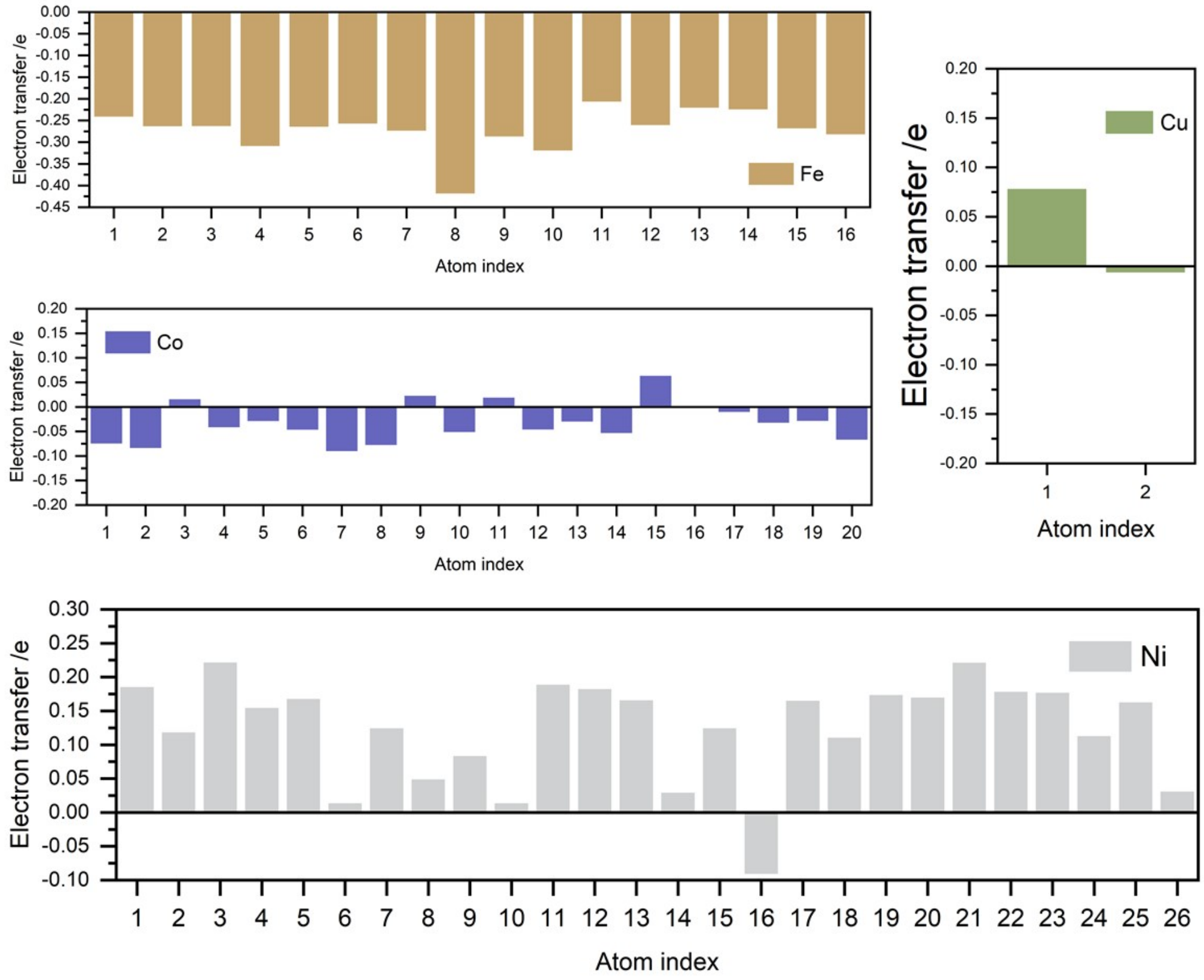

Figure S9. Bader charge analysis of the FeCoNiCu MEA system after Li_2S_6 adsorption, highlighting the strong average charge variation of Cu during long-chain polysulfide adsorption.

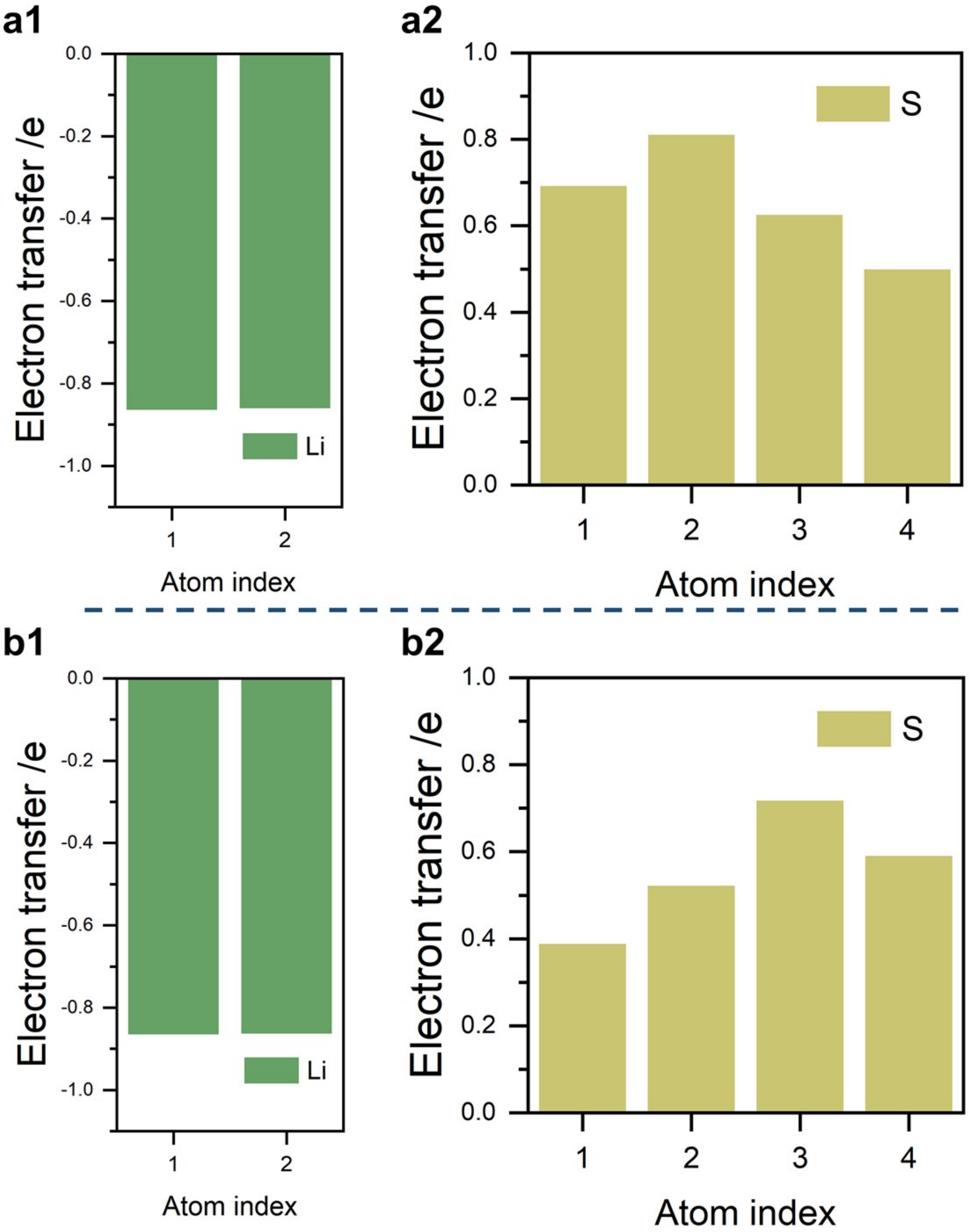

Figure S10. Bader charge analysis of Li and S atoms in adsorbed Li_2S_4 and Li_2S_6 systems, confirming charge transfer between the alloy surface and adsorbed polysulfides.

Supplementary Data Tables

The following supplementary electrochemical figures are provided as practical feasibility and contextual support for the main-text discussion. Control electrochemical data measured under different protocols are included only as contextual references and should not be interpreted as a strict same-condition performance ranking or direct proof of the independent Cu contribution.

Table S1. Matched 2C cycling metrics from the LANDdt cycle-summary export.

Sample	C_{10}	C_{100}	C_{500}	C_{1000}	Retention	Decay
FeCoNiCu@CP	739.0	663.9	498.0	284.8	38.5%	0.062% $cycle^{-1}$
FeCoNi@CP	608.0	621.8	484.3	290.0	47.7%	0.053% $cycle^{-1}$

Table S2. Three-sample Li_2S_6 symmetric-cell EIS fitting parameters.

Sample	R_s (Ω)	R_1 (Ω)	R_{ct} (Ω)	A_w	Relative RMSE (%)
FeCoNiCu@CP	24.51	9.09	10.74	1.821	1.67
FeCoNi@CP	3.01	7.45	38.23	4.884	4.48
Pristine CP	6.79	12.29	144.89	3.144	3.42

Table S3. Nominally matched FeCoNiCu@CP and FeCoNi@CP preparation conditions.

Parameter	FeCoNiCu@CP	FeCoNi@CP
0.1 M precursor aliquots	Fe/Co/Ni/Cu: 1.000 mL each	Fe/Co/Ni: 1.333 mL each
Final precursor volume	6.000 mL	6.000 mL
Drop volume per disc	60 μL	60 μL
Nominal total metal per disc	4.00 μmol	4.00 μmol
Carbon-paper disc	19 mm	19 mm
Air-plasma cleaning	100 W, both sides, 15 min	100 W, both sides, 15 min
Drying	60 °C, 20 min	60 °C, 20 min
Joule heating	30 V, 150 A, 50 ms, single pulse	30 V, 150 A, 50 ms, single pulse
Maximum set temperature	1000 °C	1000 °C

Table S4. Matched CV kinetic parameters from the second complete cycle: I_p–$v^{1/2}$ regression slopes and peak metrics at 0.10 mV s^{-1} for FeCoNiCu@CP and two FeCoNi@CP replicates.

(a) I_p–$v^{1/2}$ regression slopes [mA (mV s^{-1}) $^{-1/2}$] and linear-fit R^2 values.

Sample	Anodic slope	Anodic R^2	High-cathodic slope	High-cathodic R^2	Low-cathodic slope	Low-cathodic R^2
FeCoNiCu@CP	13.086	0.9951	5.799	0.9961	7.997	0.9926
FeCoNi@CP rep. 1	13.879	0.9905	6.981	0.9973	6.754	0.9989
FeCoNi@CP rep. 2	15.542	0.9910	7.574	0.9969	7.223	0.9997

(b) Peak potentials and peak separations at 0.10 mV s^{-1}.

Sample	E_{pa} (V)	$E_{pc\text{-}high}$ (V)	$E_{pc\text{-}low}$ (V)	ΔE_{high} (V)	ΔE_{low} (V)
FeCoNiCu@CP	2.3855	2.3546	1.9956	0.0309	0.3899
FeCoNi@CP rep. 1	2.4224	2.3725	2.0046	0.0499	0.4178
FeCoNi@CP rep. 2	2.3869	2.3710	2.0031	0.0159	0.3838

Table S5. Spin-resolved dopant d-band centers relative to E_F = 0 eV.

Fourth element	Combined center (eV)	Spin-up (eV)	Spin-down (eV)	Selected atoms
Sc	2.07	2.244	1.878	2
Ti	1.03	1.331	0.762	2
V	0.41	0.478	0.342	2
Cr	0.37	−0.669	1.328	2
Mn	−0.25	−1.639	1.206	2
Cu	−2.44	−2.551	−2.336	2
Zn	−6.58	−6.530	−6.622	2
